# Transcriptome-wide identification of the RNA-binding landscape of the chromatin-associated protein PARP1 reveals functions in RNA biogenesis

**DOI:** 10.1038/celldisc.2017.43

**Published:** 2017-11-28

**Authors:** Manana Melikishvili, Julia H Chariker, Eric C Rouchka, Yvonne N Fondufe-Mittendorf

**Affiliations:** 1 Department of Molecular and Cellular Biochemistry, University of Kentucky, Lexington, KY, USA; 2 Department of Psychological and Brain Sciences, University of Louisville, Louisville, KY, USA; 3 Kentucky Biomedical Research Infrastructure Network Bioinformatics Core, 522 East Gray Street, Louisville, KY, USA; 4 Department of Computer Engineering and Computer Science, University of Louisville, Louisville, KY, USA

**Keywords:** PAR–CLIP, PARP1, RNA-binding proteins, transcription, alternative splicing

## Abstract

Recent studies implicate Poly (ADP-ribose) polymerase 1 (PARP1) in alternative splicing regulation, and PARP1 may be an RNA-binding protein. However, detailed knowledge of RNA targets and the RNA-binding region for PARP1 are unknown. Here we report the first global study of PARP1–RNA interactions using PAR–CLIP in HeLa cells. We identified a largely overlapping set of 22 142 PARP1–RNA-binding peaks mapping to mRNAs, with 20 484 sites located in intronic regions. PARP1 preferentially bound RNA containing GC-rich sequences. Using a Bayesian model, we determined positional effects of PARP1 on regulated exon-skipping events: PARP1 binding upstream and downstream of the skipped exons generally promotes exon inclusion, whereas binding within the exon of interest and intronic regions closer to the skipped exon promotes exon skipping. Using truncation mutants, we show that removal of the Zn1Zn2 domain switches PARP1 from a DNA binder to an RNA binder. This study represents a first step into understanding the role of PARP1–RNA interaction. Continued identification and characterization of the functional interplay between PARPs and RNA may provide important insights into the role of PARPs in RNA regulation.

## Introduction

Poly (ADP-ribose) polymerase 1 (PARP1) or ADP-ribosyl transferase 1, a multifunctional nuclear protein, belongs to the PARP family of proteins. PARP1 is responsible for initiation, elongation, and branching of ADP-ribose units from donor NAD^+^ molecules onto target proteins, a process known as PARylation. The major target for PARylation is PARP1 itself, but a number of other covalently PARylated proteins have been described, including histones, chromatin remodeling proteins, and transcription factors. PARylation influences the activity of target proteins by modulating protein–nucleic acid interactions, enzymatic activity, protein–protein interactions, and/or subcellular localization.

PARP1 was first characterized as a sensor for DNA breaks [1]. Besides its DNA damage response, PARP1 plays a crucial role in regulating numerous molecular processes, such as gene transcription and chromatin remodeling [2–4]. Some of the best functional examples of PARP1 in gene regulation are its regulation of chromatin structure by PARylating histones and destabilizing nucleosomes [5–7], its competition with H1 for specific target sites [8] and/or its direct interaction with transcription factors and cofactors, such as NF-κB or the nuclear factor to activate T-cell gene expression [9–13]. PARP1 also plays critical roles in cell division. For instance, PARP1 regulates components of the mitotic apparatus, such as centromeres and centrosomes, to control microtubule organization during mitosis and chromosome segregation [10]. Taken together, these studies show that PARP1 exhibits a wide array of subcellular distributions, suggesting a broad and varied role for this protein [14, 15].

Although PARP1 has been implicated in multiple regulatory processes, one process for which the paradigm may change is its role in RNA biogenesis. First, PARP1 is known to PARylate poly (A) polymerase (PAP), inhibiting its polyadenylation activity [16], with consequences for pre-mRNA splicing regulation. Second, PARP1 binds to noncoding pRNAs to silence rDNA chromatin [17]. Third, PARP1 PARylates heterogeneous nuclear ribonucleoproteins (hnRNPs), which play important roles in pre-mRNA splicing and translation regulation [18]. Fourth, we recently identified PARP1 as an mRNA-binding protein [19, 20], providing further evidence that PARP1-/PARylation-mediated events function directly to control pre-mRNA processing. These findings serve to define PARP1 as a co-transcriptional splicing regulator [20]. One possible mechanism for this co-transcriptional function is that PARP1 acts as an adapter, bringing RNA close to chromatin [20]. In fact, a widespread association of chromatin-binding proteins with RNA was shown *in vivo*, supporting the idea of co-transcriptional RNA splicing [21].

We previously identified PARP1 as a novel RNA-binding protein (RBP) using photoactivatable-ribonucleoside-enhanced crosslinking and immunoprecipitation (PAR–CLIP). This study raised the interesting possibility that PARP1 plays crucial roles in many aspects of RNA processing to alter gene expression via regulation of mRNAs. Taken together, the identification and characterization of PARP1−mRNA interactions may provide important insights into the role of PARP1 in mRNA regulation and subsequent human disease. However, the breadth, range, and functional location of mRNA types bound by PARP1 has not been explored. In order to identify the direct RNA targets and precise binding sites of PARP1 protein *in vivo*, we again applied PAR-CLIP followed by deep sequencing (PAR-CLIP-seq). This method is known for its precise identification of binding sites resulting from T-to-C sequence conversions upon RNA–protein crosslinking. We observed that PARP1 was predominantly crosslinked to mRNAs. PAR–CLIP-binding regions contained guanine–cytosine-rich sequences, and RNA–protein interaction was further confirmed by gel mobility-shift assays. Furthermore, we narrowed down the RNA-binding region of the PARP1 protein. The enrichment of many other mRNA-binding proteins (mRBPs) among the large number of PARP1–mRNA targets suggests that PARP1 has a broad role in the regulation of many genes. A continuous identification and characterization of functional interplay between PARPs and RNA may provide important insights into the role of PARPs in RNA regulation.

## Results

### PARP1 binds distinct coding and ncRNA sequences

**Figure 1:**
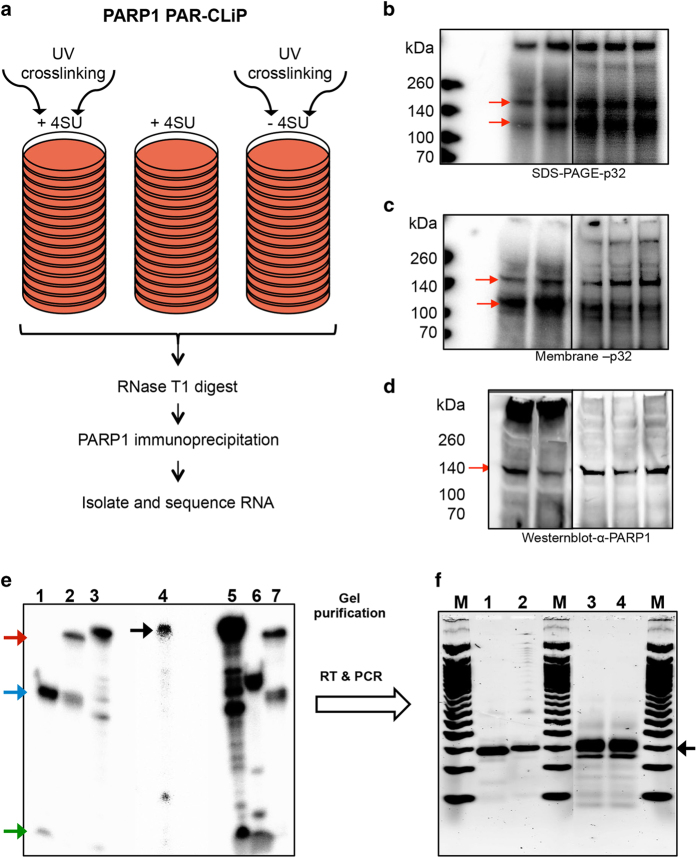
Processing of PAR-CLIP RNA samples for sequencing. (**a**) Outline of experiments. PAR-CLIP of endogenous PARP1 was performed on HeLa cells using 4-thiouridine (4SU). (**b**) PAR-CLIP samples run on an SDS PAGE gel were imaged with Typhoon. (**c**) Protein-bound samples were transferred onto a nitrocellulose membrane and exposed to a phosphoimager screen and imaged using the Typhoon. (**d**) The same membrane in **a** was probed for the presence of PARP1-bound RNAs using an antibody to PARP1. Red arrows (**b–d**) indicate PARP1 bound-RNA—one at ~140 kDa and a shorter fragment at ~100 kDa. As the antibody recognizes the larger fragment, we considered this as the full-length protein. The lower band could be a proteolytic fragment as determined by mass spectrometry, lacking the N terminus, and rendering it undetectable by the antibody raised against the N-terminal domain of the protein. (**e**) Processing of PARP1-bound RNAs for sequencing. A representative denaturing (8 M urea) polyacrylamide gel showing the different steps of adapter ligation to RNA samples. PARP1-bound RNAs were eluted from membrane in **c**, deproteinized, ligated to 3′ and 5′ adapters (lane 4). Lanes 1 and 6 are control 19-mer and 24-mer labeled RNAs, ligated to 3′ adapter. These ligated 3′ adapter control RNAs were further ligated to 5′ adapters (lanes 2 and 7). The green arrow indicates unligated 19-mer and 24-mer RNAs (Lanes 1 and 6, respectively); the blue arrow indicates 3′-adapter ligated control RNAs; the red arrow indicates 3′ adapter and 5′-adapter ligated control RNAs. These controls were used to test the ligation efficiency of our samples. The black arrow (lane 4) indicates 3′ and 5′ adapter ligated PARP1-bound RNA samples. (**f**) Adapter-ligated samples were subjected to limited PCR amplification. Lanes 1 and 2 show 3′ and 5′ ligated 19-mer and 24-mer control RNAs converted to cDNA and PCR-amplified. Lanes 3 and 4 are the PARP1-bound RNAs subjected to cDNA conversion and PCR amplification. The black arrow shows the PCR products used for sequencing.

In our previous experiments we established for the first time that PARP1 binds to RNA *in vivo* [20]. In the present study, we expanded on previous studies [20, 22] to identify PARP1–RNA targets utilizing the PAR-CLIP-seq method [23–26] (Figure 1a) in human HeLa cells. Following UV crosslinking, PARP1-bound RNAs were immunoprecipitated under stringent conditions. Radiolabeled PARP1-bound RNA complexes were separated by NuPAGE and observed using a Phosphorimager (Figure 1b). To ensure that only PARP1 protein-bound RNAs were used for further analysis, gels were transferred onto nitrocellulose membranes, visualized by autoradiography (Figure 1c), and the presence of PARP1-bound RNAs was confirmed by western blot analysis (Figure 1d). The results from these experiments demonstrate the robustness and specificity of the PARP1–RNA complexes identified by PAR-CLIP (Figure 1).

In the analysis of the phosphorimages of the radiolabeled PARP1–RNA complexes, we observed two major bands, one migrating at ~100 kDa and the other migrating at ~140 kDa. This ~140-kDa band based on the estimation from the protein standard we used is indeed PARP1 (Using other protein standards, this band runs according to PARP1’s predicted molecular weight of ~116 KDa—see Supplementary Materials and Methods). Indeed, this band was later confirmed by western blot analysis as PARP1-bound RNA (Figure 1d). Stringent digest with RNase T1 resolved the PARP1–RNA bands to within the estimated molecular weight of PARP1 ~140 kDa (Supplementary Figure S1a). The 100-kDa band contains cleaved PARP1 as identified with antibody that recognizes the c-terminal domain of PARP1 (data not shown). In addition to these two bands, we also observed signals from a higher molecular weight complex (>260 kDa), possibly due to larger complexes that did not migrate into the gel (Supplementary Figure 1a). We suspect that this band likely represents other abundant RNA binding near PARP1-binding sites, or PARP1 crosslinked to longer target RNA segments [27] (Figure 1b–d). This interpretation is reasonable, given that similar trends have been observed with other RNA-binding proteins [28].

To validate the specificity of PARP1–RNA binding, we performed several control experiments. (1) A control PAR-CLIP experiment using nonspecific antibodies (IgG) to precipitate RNA complexes failed to detect any RNA (not shown). (2) Cells not treated with thiouridine or non-crosslinked cells failed to immunoprecipitate a significant amount of PARP1-bound RNA (Supplementary Figure S1b, lanes 2 and 3, respectively), although PARP1 protein remained efficiently precipitated as determined by western blot analysis of the immunoprecipitated complexes (Supplementary Figure S1c, lane 5, bottom; Supplementary Figure 1d, Lanes 3 and 5, bottom). (3) Experiments with stringent RNaseA treatments eliminated the PARP1–RNA bands (Supplementary Figure S1b, Lanes 6 and 7, Supplementary Figure S1e). (4) Knockdown of PARP1 abolished the PARP1–RNA band (Supplementary Figure S1c, lane 6). (5) Lastly, treatment of cells with PJ34 (PARylation inhibitor) for 1 or 24 h did not change the PARP1–RNA-binding profile (Supplementary Figure S1c, lanes 3 and 4), suggesting that this binding is specific for PARP1 and not PAR.

After confirming PARP1–RNA binding, the PARP1–RNA complexes were cut from the membrane, eluted, deproteinized, purified, and ligated to adapters (Figure 1e). The resulting ligated RNAs were converted to cDNA followed by limited PCR amplification experiments (Figure 1f and Supplementary Figure S2a). Initially, these PCR fragments were cloned into TOPO-blunt vector, checked for correct insert size by restriction enzyme digest (Supplementary Figure S2b), and Sanger-sequenced. From these pilot experiments, the mean fragment length was 21 nucleotides (from the main 140 kDa PARP1–RNA band), 31 nucleotides (from the 200 kDa–PARP1–RNA fragment), and 7 nucleotides from (70 kDa–PARP1–RNA band; Supplementary Figure S2c). For subsequent studies, only the bands resulting from the main PARP1-bound RNA bands (~140 kDa) were used. Seven biological replica experiments were performed, barcoded, and pooled for sequencing using PE Illumina sequencing on a HiSeq 2500. From the various biological replicate experiments, we obtained 0.9–97×10^6^ reads after sequencing (Supplementary Table S1). These sequences were subsequently trimmed from adapter sequences yielding a total of 0.6–39×10^6^ unique reads, 47% of which mapped to the human genome (hg38) allowing 0–2 mismatches.

**Figure 2:**
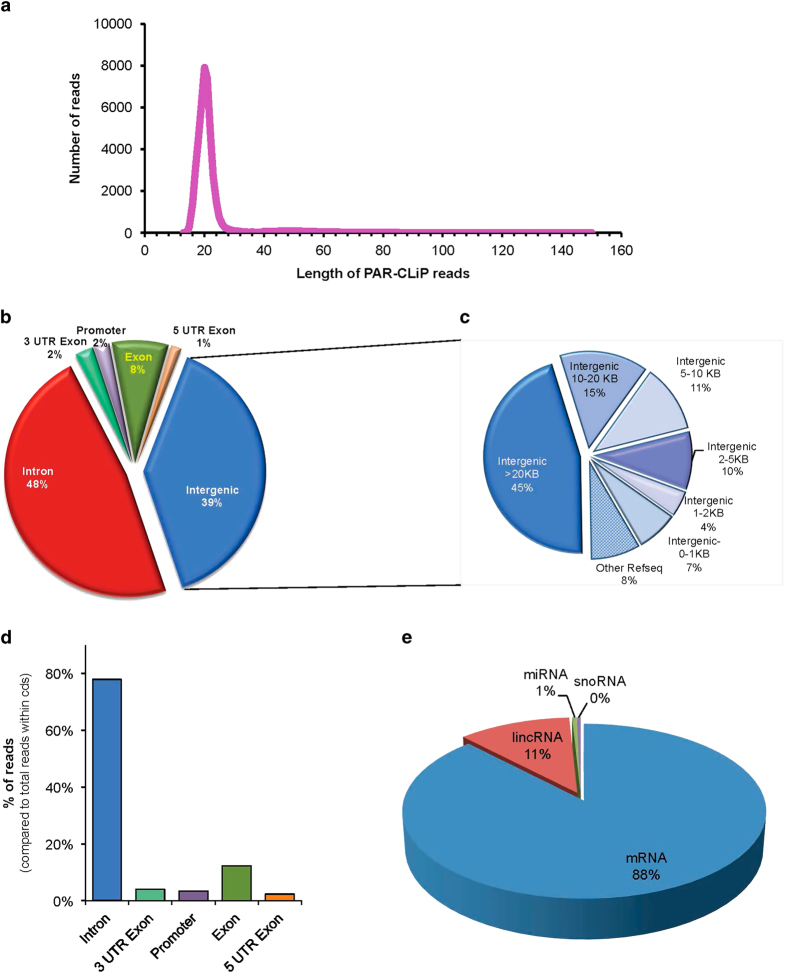
PARP1 RNA binding characteristics. Analyses of PARP1 PAR-CLIP-tags. (**a**) Fragment length distribution of the PARP-CLIP-reads. Insert Table 1 shows the length and number of PARP1 PAR-CLIP sequences. (**b**) The pie chart shows PARP1-PAR-CLIP peaks. Most PARP1-CLIP tags mapped to intronic regions, indicating PARP1 binding to mRNA. The intron enrichment is consistent with previous reports that PARP1 binds mainly nascent transcripts. (**c**) Further analyses of the intergenic regions show other possible regions that are regulatory. (**d**) Distribution of PARP1 PAR-CLIP tags within gene regions. (**e**) Distribution of PARP1-PAR-CLIP reads within different types of RNA.

Next, we grouped them by overlaps using the PARalyzer software [29]. The identified segments of RNA represented peaks of T-to-C conversion (binding sites), with a mean length of 21 nucleotides (mean and mode of 20 nt) from uniquely aligned T-to-C reconciled reads (Figure 2a and Table 1). Groups of overlapping PAR-CLIP sequence reads were considered binding sites if they (1) passed thresholds of ≥0.25 for T-to-C conversion frequency, (2) contained more than five reads with T-to-C conversion (one mismatch maximum allowed per read), and (3) showed at least two independent T-to-C conversions. Biological replicates, although with different sequencing depth, showed similar binding patterns (Supplementary Figure S3).

To identify PARP1–RNA target sites, we analyzed the distribution of PAR-CLIP tags in the human genome by defining six regions (exon, introns, promoter, 5′ UTR, 3′ UTR, and intergenic regions). The distribution of binding sites across individual transcripts provided insights into PARP1 targeting. Approximately 48% of PAR-CLIP peak tags (see Materials and Methods) mapped to introns, ~8% mapped to exons, 2% to promoter regions, 2% to 3′ UTR, 1% to 5′ UTR, and 39% mapped to intergenic regions (Figure 2b). The over-representation of intronic PAR-CLIP reads indicates that PARP1 binds pre-mRNAs (nascent transcripts) and is consistent with our hypothesis that PARP1 plays a role in pre-mRNA splicing and processing. On the other hand, the observation of a high percentage of PARP1-PAR-CLIP reads to intergenic regions suggests the possibility that these PAR-CLIP tags may correspond to previously unidentified isoforms of genes with alternative terminal exons. To test this idea, we carried out two types of analyses. First, we examined the distance between intergenic clusters and neighboring RefSeq genes. An exponential increase in the cumulative number of tags within 10 kb downstream of known stop codons compared to linear increases beyond 10 kb was detected. For instance, 39% of these intergenic peaks mapped within 10 kb of the nearest stop or start codon, respectively (Figure 2c and Supplementary Table S2). This suggests that in addition to binding known 3′ UTRs (Figure 2b), PARP1 binds to unannotated 3′ UTR extensions of known genes (Supplementary Figure S4). Second, we asked whether the remaining intergenic reads map to genes annotated in other reference genomes, as determined from the 'RefSeq Other' track in the UCSC genome browser. We observed that 8% mapped to genes annotated within other RefSeq genomes. These analyses show that by doing a more detailed analyses only ~18% (45% of the initial 39% intergenic reads as shown in Figure 2b) of PARP1-PAR-CLIP tags map to intergenic regions (Figure 2c and Supplementary Figure S4).

### mRNA is the major species bound by PARP1

Next, we analyzed the distribution of PARP1-PAR-CLIP reads in coding regions. This analysis showed that ~78% of the reads mapped to introns (Figure 2d), raising the possibility that PARP1 contributes to the recognition of specific intronically encoded RNAs such as mRNAs, microRNAs, small nuclear RNAs, and heterogenous RNAs, and influences the rates of various competing RNA processing steps. To examine this, we analyzed the types of RNAs bound by PARP1 from the PAR-CLIP data. Our analyses show that most of the PAR-CLIP peaks were within mRNAs (88%) compared to the other RNA types, demonstrating that mRNA is the major substrate of the PARP1–RNA complex. On the other hand, crosslink sites were also detected in different classes of RNAs: 2 870 peaks (or 11% of total RNAs bound) in long intergenic noncoding RNAs, 124 peaks (or 1% of the total RNAs bound) in microRNAs, and 88 peaks within small nuclear RNAs (Figure 2e and Supplementary Table S3). These results suggest possible novel functions for PARP1 in the regulation of the metabolism of other RNAs as well.

As an alternative method to validate these binding sites, we performed formaldehyde-crosslink RNA immunoprecipitation with nuclear extracts [30]. Enrichment of candidate RNAs was similarly observed using this method (Supplementary Figure S5). Combined, these data support the specificity of PARP1-PAR-CLIP-seq and suggest that our observed interactions are indeed interactions between PARP1 and RNA.

### RNA motifs bound by PARP1 

**Figure 3:**
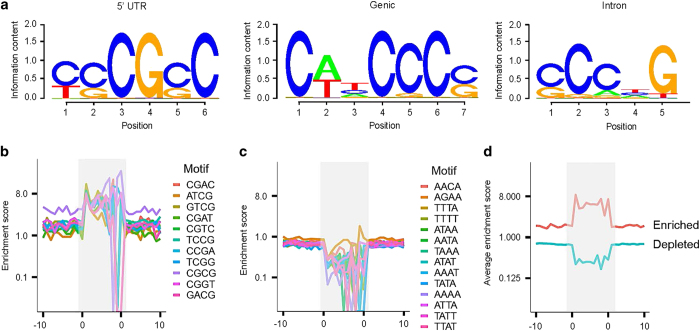
Sequence-binding characteristics of PARP1-bound RNAs. (**a**) Logo plots of some of the motifs found in the various PARP1-binding regions as determined by cERMIT. (**b**) Enriched 4-mer motifs found within percentile regions of the PARP1-binding site (gray) along with their enrichment scores in the flanking upstream and downstream regions. (**c**) Depleted 4-mer motifs within the PARP1-binding site (gray) along with their enrichment scores in the flanking upstream and downstream regions. The enrichment scores for **b**, **c** are determined by the log2 odds score of the frequency of the motif in the region versus the frequency of the motif in the genome. (**d**) Average enrichment score for enriched and depleted motifs from (**b**, **c**), respectively.

We next asked whether PARP1 binds to a particular RNA sequence motif. For that, we applied cERMIT [31] to define the *in vivo* RNA recognition element for PARP1. The three highest-scoring motifs were generally GC-rich (Figure 3a); this nucleotide composition was observed regardless of the mRNA region of the identified PAR-CLIP tags (Figure 3a). Failure to determine a highly conserved binding motif prompted us to use an unbiased k-mer approach to determine the enrichment of specific sequences within PAR-CLIP data. For this, the 2-nt PARP1-PAR-CLIP data set surrounding the crosslink sites was compared to the genome as a whole to identify k-mers enriched in PARP1-PAR-CLIP reads. Our choice of k-mers allowed us to detect smaller localized signals than cERMIT, which begins with 5-mer seed regions. Starting with 3-mers, we observed an enrichment of GC-rich 3-mers (data not shown). However, as RNA recognition elements are typically longer than 3-mers, we performed further analyses using 4-mers. Again, this analysis showed an enrichment of GC-rich 4-mers (Figure 3b), whereas AT-rich 4-mers were depleted (Figure 3c and d). We repeated the analyses with 6-mers and 8-mers, and clearly the enriched k-mers were GC-rich k-mers, although these longer GC-rich are interspersed by AT k-mers (Supplementary Figure S6). Our data show that PARP1 protein RNA-binding sites were comparatively GC-rich, suggesting a tolerance for these GC-rich residues, whereas AT-rich residues were relatively less well tolerated. This information is of interest as during PAR-CLIP experiments G-containing sequences are normally trimmed by RNase T1, and the only way for these guanosines to survive this cleavage is if they are protected by direct binding of the PARP1 or by stable RNA secondary structure [32]. Our results therefore suggest that PARP1 binds to GC-rich regions and protects these G-rich regions from RNase T1 cleavage.

### Splicing and gene expression changes in response to PARP1 loss

**Figure 4:**
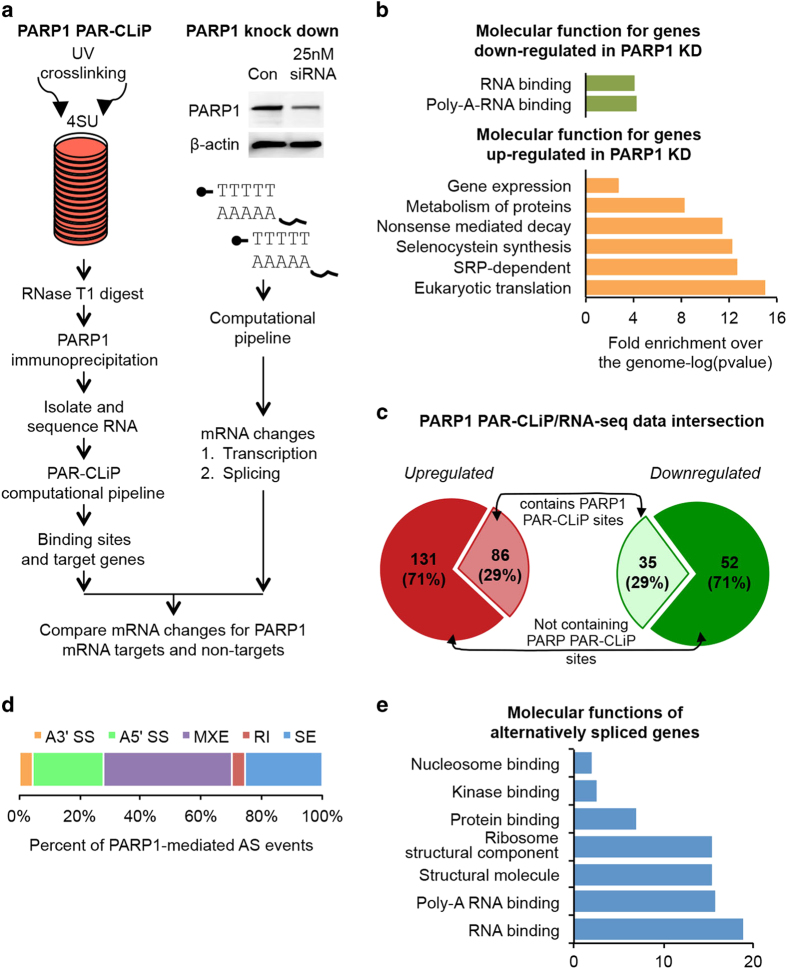
Loss of PARP1 targeted transcriptional and splicing regulation. (**a**) Experimental and computational workflow for analyzing the intersection of RNAs bound by PARP1 and RNAs whose expression is mediated by PARP1. Left side shows PAR-CLIP workflow, whereas the right side shows RNA-seq workflow after PARP1 depletion (**b**). Gene Ontology (Molecular function terms) for genes with significantly changed expression in PARP1 knockdown cells. (**c**) Proportion of genes overlapping in combined RNA-seq/PARP1-PAR-CLIP data sets. (**d**) Percentage of the alternative splicing events identified as PARP1 mediated with PARP1 knockdown: alternative 3′ splice site (orange); alternative 5′ splice site (green); mutually exclusive exons (purple); retained intron (pink); skipped exon (blue). (**e**) Gene Ontology analysis (molecular function terms) of the genes identified as PARP1-regulated at the splicing level.

To test whether transcripts bound by PARP1 are affected upon PARP1 depletion, we determined the global patterns of PARP1-dependent transcription/splicing changes. For this, cells were transfected with ONTARGETplus short interfering RNA (siRNA) targeting PARP1 and for control experiments with non-targeting siRNAs. Depletion of PARP1 protein was confirmed by western blot analyses, which showed an ~70% reduction in PARP1 protein levels in the knockdown cells (Figure 4a). Total RNA was isolated from control non-targeting siRNA and PARP1 knockdown (KD) cells, and poly(A)-selected mRNA sequencing was performed on the Illumina platform. Biological replicas from RNA-seq showed high Pearson correlation (Supplementary Table S4), allowing pooling of samples for further analyses. First, we measured changes in gene expression at the transcript level due to PARP1 knockdown. We identified 217 significantly upregulated and 81 downregulated genes, including *PARP1* (using a cutoff of twofold expression and *P-*value of 0.05 versus non-targeting control; Supplementary Table S5). GO analysis using Gene Set enrichment analysis (GSEA) showed that the top biological processes targeted by the genes upregulated in PARP1 KD cells are NMD, translation, protein metabolism, selanocysteine synthesis, and gene expression. Genes that were downregulated in PARP1 knockdown cells are involved in RNA-binding and poly-A-RNA-binding using GSEA (Figure 4b). We next compared PARP1 RNA targets to genes affected by PARP1 knockdown, and did not observe any meaningful correlation between genes that were bound and trends in gene expression changes. Nevertheless, we observed that ~29% of genes transcripts affected by PARP1 knockdown were also bound by PARP1 in our PAR-CLIP analysis (Figure 4c).

Our previous study in *Drosophila* cells suggested that PARP1 plays a role in alternative splicing regulation [20]. In order to assess the effect of PARP1 in splicing, we also analyzed the RNA-seq data for differential alternative splicing events. Using stringent criteria to identify changes in alternative splicing events, we showed that PARP1 depletion resulted in changes in alternative splicing for 791 genes. These changed events included mutually exclusive exons (42.4%), skipped exon (25.6%), retained intron (4.2%), alternative 5′ splice site (23.5%), and alternative 3′ splice site (4.4%; Figure 4d). We validated some of these changes in alternative splicing due to PARP1 depletion using qRT-PCR (Supplementary Figure S7). The number of alternatively spliced genes are slightly lower than those observed in our previous studies with *Drosophila*, where we observed many more changes in alternative splicing [20]. We attribute this low number to possible redundancy with other PARP proteins in humans. GO molecular function terms as determined using GSEA for the targeted alternative spliced genes include nucleosome binding, Poly-A-binding, and RNA binding (Figure 4e).

### Positional effects of PARP1 in splicing regulation

**Figure 5:**
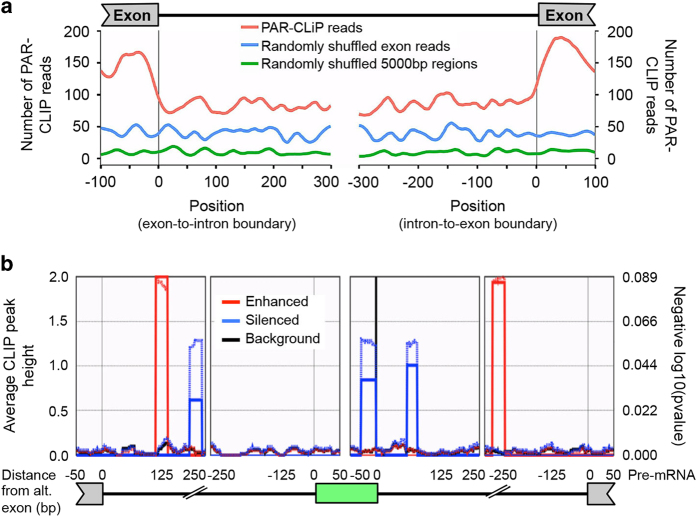
Positional analysis of PARP1-PAR-CLIP tags, with respect to splicing functions. (**a**) The number of PAR-CLIP sequences covering exon boundaries (red) compared to the number of sequences covering randomly shuffled exons (blue) and randomly selected 5 000-bp regions (green). (**b**) Maps for PARP1-PAR-CLIP read enrichment for the skipped exon events from RNA-seq of PARP1 knockdown and control (non-targeting siRNA) experiments are shown for enhanced (red) and silenced (blue) splicing events. Solid lines represent the peak quality score (peak height) as scaled on the left. Dotted lines represent the significance score (*P*-value) as scaled on the right. The level of significance was determined by comparison to a ‘Background set’ of 32 114 of non-impacted alternative exons (rMATs FDR >50%) in expressed genes (FPKM>5.0). Only events showing *P*≤0.05, FDR≤0.05, and a minimum inclusion level difference ≥0.1 were considered. The green box indicates the PARP1-regulated exon.

To extend the analysis of the role of PARP1 in alternative splicing, we averaged the presence of PARP1 PAR-CLIP reads along all exon/intron and intron/exon boundaries, representing 3′ and 5′ splice sites, respectively. PARP1 binds uniformly within introns, whereas its binding is enriched at the ends of exons—specifically within 50 nucleotides upstream of start of the exon and 50 nucleotides downstream of the end of the exon (Figure 5a). The observed exon bias reflects the distribution of binding sequences within target RNAs and suggests that PARP1 binds mRNA. Although we had observed PARP1 PAR-CLIP reads in introns (Figure 2b), the density of these reads at exon–intron boundaries suggests a functional role of PARP1 in demarcating exons. Thus, the binding of PARP1 preferentially at exonic sequences, especially upstream of 5′ and 3′ of splice sites, is consistent with the model that proteins that regulate splicing bind pre-mRNA at functional regions.

To better understand the impact of PARP1 in splicing, we combined PAR-CLIP data with the analysis of splicing profiles upon PARP1 depletion to determine the position-dependent regulatory effects of PARP1–RNA interactions. To this end, we analyzed the rMATS outputs for skipped exon events using the bioinformatics software rMAPS [33], which systematically generates RNA maps for the identification of position-dependent effects of RNA-binding proteins. The rMAPS program is extremely useful for the computational detection of binding sites around differential alternative splicing events for over 100 of known RBPs. Using the rMAPS-based analysis (with default parameters), along with the list of all PARP1 PAR-CLIP peaks and detected skipped exon events, we identified binding patterns of PARP1 within the *PARP1-*dependent alternatively spliced exons (Figure 5b). Restricting the analyses to only significant exon-skipping splice events, we found that for those enhanced and included exons, there is a significant PARP1 binding occurring about 125 bp downstream of the adjacent 5′ exon, and binding occurring about 250 bp upstream of the adjacent 3′ exon (peaks in red). If the exon is excluded, there is a significant binding of PARP1 within the exon itself (in blue) as well as within the upstream and downstream introns. Although it is possible that factors related to translational efficiency and/or RNA stability may affect the regulatory landscape of PARP1-responsive splicing events, the differential expression of the PARP1 together with the enrichment of PARP1-binding and its positional enrichment relative to the regulated exons suggests that many or most of the identified skipped exon splicing events are likely direct targets.

### Biochemical characterization of PARP1 protein–RNA-binding sites

**Figure 6:**
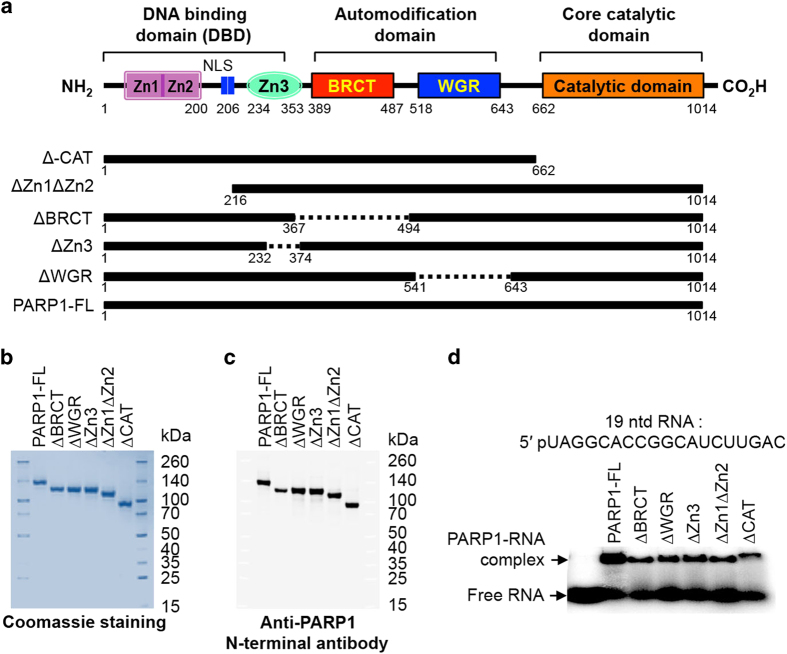
PARP1–RNA domain interactions. (**a**) Schematic structure of PARP1 showing the various functional domains important in PARP1 activation, localization, and activity. The first three N-terminal domains (residues 1–353) are zinc-finger DNA-binding domains with distinct functions in PARP1 DNA nick-mediated activation. The central, auto-modification region of PARP1 contains a BRCT domain (residues 389–487), as well as flanking residues that serve as sites of auto-ADP ribosylation. Adjacent to the BRCT domain is a WGR domain (residues 518–643) followed by the catalytic domain (residues 662–1 014), which possesses activities related to the ADP-ribose adduct formation, elongation, and branching activity. Below are the various PARP1 constructs created to test the RNA-binding activity of PARP1. (**b**) Coomassie gel staining of purified recombinant full-length PARP1 and the truncated PARP1 proteins. (**c**) Western blot analysis of PARP1 and truncated PARP1 proteins. (**d**) Gel shift assays showing the binding of RNA by various proteins, PARP1 full-length, and truncations.

PARP1 encompasses several functional domains: three zinc-finger domains (Zn1, 2, and 3), a nuclear localization signal region, a breast cancer suppressor protein-1 domain (BRCT), a WGR domain (automodification domain), and the catalytic PARP domain (Figure 6a). To begin to understand PARP1-RNA binding, we purified recombinant full-length human PARP1 (PARP1-FL) and truncated mutants lacking the C-terminal catalytic active site (ΔCAT), the DBD—the first two zinc fingers (ΔZn1Zn2); the third zinc-finger domain (ΔZn3), the automodification domain (ΔWGR), or the protein–protein interaction domain (ΔBRCT domain) from bacterial cells (Figure 6b). Their presence was confirmed through western blot analyses using PARP1 antibody (lanes 1–6, respectively; Figure 6c) and their proper folding confirmed using circular dichroism spectroscopy analyses (Supplementary Figure S8a). We addressed whether PARP1–RNA direct binding is dependent on other factors, such as contaminating DNA and/or PARP1 PARylation. First, recombinant PARP1-FL was incubated with a radiolabeled synthetic 19-mer ssRNA (chrom15: 53554024-53554044) corresponding to one of the binding sites identified by PAR–CLIP. The protein–RNA complexes were then resolved on a native polyacrylamide gel (Figure 6d and Supplementary Figure S8b). A supershift corresponding to PARP1–RNA complex was observed (Supplementary Figure S8b, lane 2). Second, the PARP1–RNA complex was treated either DNase1 or RNaseA, confirming that RNA is the nucleotide species bound by PARP1 as DNase1 treatment did not change the binding profile but RNaseA completely digested the RNA (Supplementary Figure S8b, lanes 5 and 6, respectively). In addition, treatment of PARP1 with PJ34 did not inhibit PARP1 binding to RNA (Supplementary Figure S8b, lane 4), whereas PARylation of PARP1 by NAD+ abolished its RNA-binding (Supplementary Figure 8b, Lane 3), indicating that PARP1–RNA binding is due to PARP1 and not PAR. As a control, RNA was incubated with increasing amounts of bovine serum albumin and no significant shift in RNA mobility was observed (data not shown).

**Figure 7:**
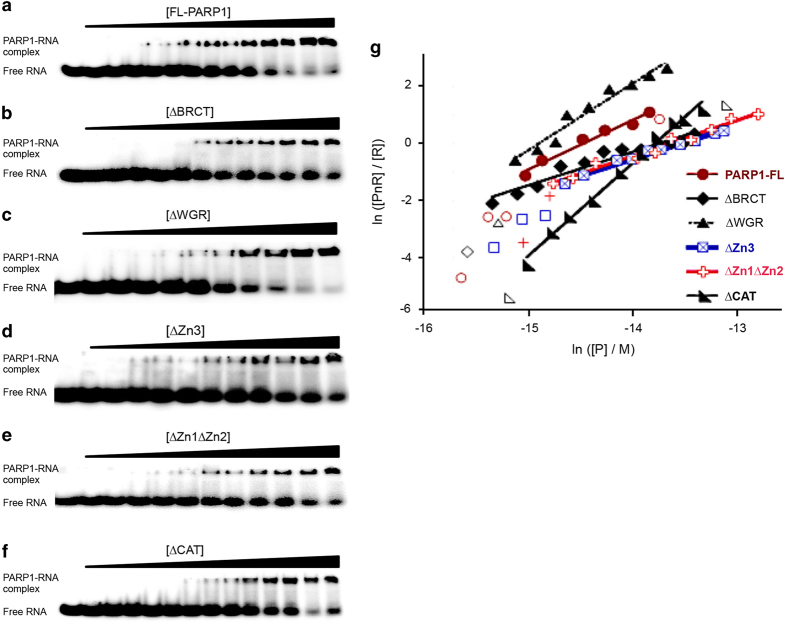
Gel-shift assays for the binding of PARP1 proteins to single-stranded 19-mer RNA. (**a**–**f**) Full-length PARP1 proteins and the truncated mutants bind single-stranded 19-mer RNA. In these experiments, RNA (0.05 μM) was incubated with increasing concentrations of the six different [PARPs] (0–2.5 μM from left to right). The lower bands indicate free RNA and the upper bands are PARP1–RNA protein complexes. (**g**) Associated binding isotherm analysis. The slope of each isotherm is a measure of stoichiometry for the individual PARP1 protein binding to the RNA. Values of *n* and *K* are summarized in Table 2.

We next asked which domain of PARP1 is required for its PARP1–RNA binding. EMSA was performed using PARP1-FL as well as truncated mutants by individually incubating them with the radiolabeled synthetic 19 mer RNA (as above; Figure 6d). As seen previously, discrete shifted bands corresponding to PARP1–RNA complexes were observed for all the proteins tested. We then determined the binding affinities of PARP1-FL and mutants to RNA by performing EMSA, incubating 0.05 μM radiolabeled 19-nt RNA with increasing concentrations (0–2.5 μM) of PARP1-FL or truncated proteins (Figure 7a–f). The fraction bound to total RNA as a function of increased protein concentration for each protein was used to calculate the affinity of that particular protein for RNA (Figure 7g and Supplementary Figure S9). Interestingly, these proteins bind with different stoichiometry, and this difference in binding stoichiometry was taken into account when calculating the affinity constants−*K*_assoc_ (Table 2). These results show only a two- to threefold difference in affinity to RNA between the PARP1 proteins—with PARP1-FL having the highest affinity, whereas ΔZn3 showed the lowest affinity, followed by ΔZn1ΔZn2 (Table 2). These data are in line with previous studies that showed that PARP1 binds RNA via its zinc-finger 3 domain [34]. Interestingly, deletion of another region previously implicated in binding RNA (WGR) did not significantly change the affinity from that of the PARP1-FL. Similar binding affinity results were obtained using RNAs of different lengths (20 and 24 nt; Supplementary Table S6). At first surprising, similar small differences in affinity have also been recorded for the binding of these constructs to DNA [35], although PARP1 is a well-known DNA-binding protein. These previous results hypothesized that all the domains of PARP1 contribute to its DNA-binding interactions. We believe that a similar scenario is occurring with PARP1 binding to RNA.

**Figure 8:**
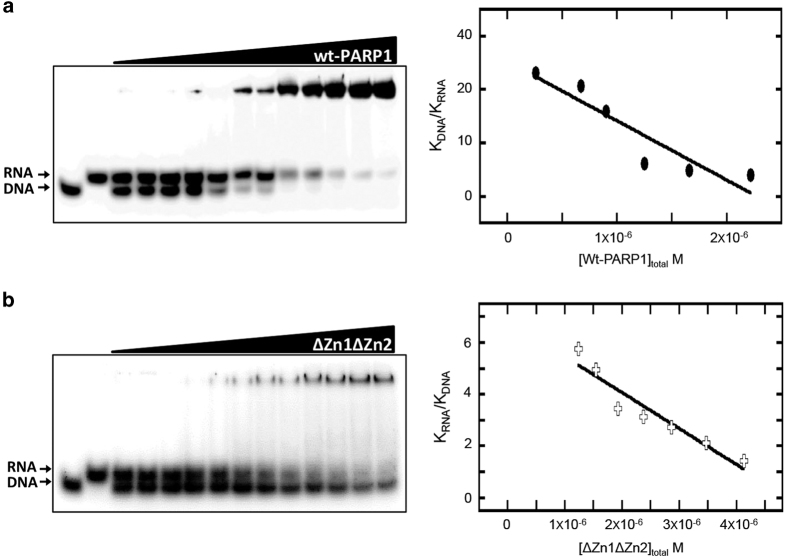
In the absence of Zn1Zn2, PARP1 preferentially binds RNA *in vitro*. PARP1 was incubated with radiolabeled ssRNA and radiolabeled ssDNA of the same sequence in the same reaction. (**a**) Radiolabeled 19-ntd ssRNA and ssDNA were incubated with increasing concentrations of PARP1-FL. Left, EMSA of PARP1–DNA and PARP1–RNA complex formation with increasing concentrations of PARP1-FL. The DNA band disappears faster than the RNA band. Right, graph depicts the relationship between radiolabeled DNA/RNA and increasing concentration of recombinant PARP1 in the same binding reaction. (**b**) Radiolabeled 19-ntd ssRNA and ssDNA were incubated with increasing concentrations of ΔZn1ΔZn2. Left: EMSA analyses showing the formation of ΔZn1ΔZn2–DNA and ΔZn1ΔZn2–RNA complexes with increasing concentrations of ΔZn1ΔZn2. In the case of ΔZn1ΔZn2, the RNA band disappears faster than the DNA. Right, graph depicts the relationship between radiolabeled DNA/RNA and increasing concentration of recombinant ΔZn1ΔZn2 in the same binding reaction. Because of the inability to differentiate PARP1–DNA complexes from PARP1–RNA complexes on the gel, the rate of disappearance of the DNA and RNA bands was used as a proxy to measure the affinity of PARP1 for either DNA or RNA.

Following on these results, we examined the possibility that RNA activates PARP1 and showed that, just like DNA, RNA activates PARP1, albeit at a lower extent (Supplementary Figure S10). Finally, we performed a competition assay to test whether PARP1 preferentially binds DNA to RNA. Equal concentration of radiolabeled 19-mer RNA and radiolabeled ssDNA of the same sequence was incubated together with increasing concentrations of the different PARP1 constructs. As the ssRNA and ssDNA of the same sequence run with different gel mobility, it allowed us to quantify the disappearance of the RNA and DNA in the presence of these recombinant PARP1 proteins. This analysis revealed that PARP1-FL had a 25-fold affinity to DNA than RNA (Figure 8a for PARP1-FL). A similar result was observed with the other constructs (Table 3) except for the ΔZn1ΔZn2 mutant. This mutant switched PARP1’s binding preference from DNA to RNA, with a sevenfold preference for RNA to DNA (Figure 8b and Table 3). These results indicate that, once the Zn1Zn2 site is unavailable, PARP1 preferentially binds RNA and suggest that the DNA binding is different from the site needed to bind RNA.

## Discussion

The transcriptome analysis performed here by high-throughput PAR-CLIP sequencing provides new insights into the endogenous RNA targets of *PARP1*. We found that PARP1 binds RNA *in vivo* (Figure 1). We also observed that, whereas the main target of PARP1-RNA binding *in vivo* is mRNA, it also binds other non-coding RNAs (Figure 2), suggestive of a functional role of PARP1 in their regulation. Within mRNAs, we find that PARP1 associates mainly with intronic sequences (Figure 2). However, since introns are very long and PARP1–RNA targets could target different regions of a particular intron, we also analyzed the density of the reads at functional splice sites. Our results show that there is a high density of PARP1–RNA binding at exon–intron boundaries and intron-exon boundaries (Figure 5). These results could suggest that PARP1 demarcates exons. Interestingly, we previously had showed that PARP1 binds GC-rich nucleosomes at exon boundaries [20]. It is therefore logical to assume that it binds to similar regions on chromatin as well as on RNA, possibly by recognizing specific sequences or structures on DNA and/or RNA. However, additional studies are needed to determine the structural implications of PARP1 binding. We further combined the PAR-CLIP-seq analysis with full transcriptome-wide analysis of gene expression and splicing changes upon PARP1 depletion. Combining PAR-CLIP and RNA-seq data allowed us to draw a PARP1 RNA map, which suggested that the binding of PARP1 on exons and in intronic regions immediately surrounding the regulated skipped exon leads to silencing of the downstream exon. PARP1 binding to introns further upstream and downstream of the skipped exon enhances exon inclusion (Figure 5b). The high distribution of PARP1 in introns (Figure 2) enhances the idea of a regulatory role of PARP1 in splicing, as intronic-binding proteins such as HNRNPU [36], HNRNPH1 [37], and HUR [38] have been implicated in splicing decisions. Under this scenario, the binding of PARP1 to intronic sequences mediates splicing; however, it can also remain associated with the mature mRNAs to help in other post-transcriptional mRNA processes. This seems to be occurring, as we observe a high PARP1 PAR-CLIP read density, at the ends of exons (exon–intron and intron–exon boundaries depicting 3′ and 5′ splice sites, respectively), and is in line with other intron-binding proteins [39]. Noteworthy is the fact that proteins that bind at exons interact with the RNA after transcription and initial RNA processing, whereas the intron binders are present during transcription [21], thus supporting their role in co-transcriptional splicing. However, because of the low CLIP efficiency (only ~1% of transcripts are crosslinked), it is difficult to distinguish whether the PARP1–RNA interactions are on pre-mRNA transcripts or whether a subset of these mRNAs is subsequently processed (in either alternative exons or poly (A) sites). On the other hand, our RNA map of PARP1 binding (Figure 5b) provides a functional landscape of significantly skipped alternative splicing regulation by PARP1 that can be used in future studies to further characterize the regulation of AS by PARP1. PARP1 could be modulating splicing decisions through two mutually non-exclusive mechanisms: (i) maintaining a chromatin structure that affects RNA polymerase kinetics and/or (ii) recruiting and PARylating splicing factors to splice sites on nascent mRNAs while bound onto chromatin.

PARP1 has been implicated in many cellular processes. In this study, we focused on the observation that PARP1 is involved in splicing regulation [20]. The means by which PARP1 regulates alternative splicing is still unknown. Earlier understanding of gene expression regulation suggested that DNA-binding proteins responded to sequence composition and chromatin context to promote transcription of RNA [40, 41]. RNA-binding proteins (RBPs) then bind these nascent transcripts to direct mRNA splicing, stability, localization, and translation [42, 43]. However, recent advances profiling nucleic acid–protein interactions find that many DNA-binding proteins also associate with RNA to modulate both transcriptional and post-transcriptional outcomes [19, 44–46], blurring this long-standing dogma for gene regulation. The results presented here also find that PARP1, a well-known DNA/chromatin-binding factor, binds RNA, adding to this growing list of proteins interacting with both DNA and RNA to affect gene regulation. Our study further suggests that PARP1 binding to RNA may regulate gene splicing and/or generally different levels of RNA biogenesis. Collectively, these studies suggest a more intertwined gene regulatory network (transcription and splicing) than had been previously appreciated.

Indeed, it is now known that splicing is tightly integrated with gene expression [47, 48], with splicing controlling gene expression via nonsense-mediated [49] or spliceosome-mediated [50] decay pathways. Unspliced and partially spliced transcripts can be deleterious for the cell [51, 52] and several quality-control pathways exist to degrade these faulty transcripts. The first and main line of protection (degradation of these faulty transcripts) is through the nuclear exosome process [53–55]. If this fails, a second line of defense occurs via cytoplasmic surveillance pathways [52], leading to cytoplasmic degradation. This can be triggered in two ways—the nonsense-mediated decay (NMD) pathway that recognizes premature stop codons [56, 57] or by the non-stop decay pathway that identifies transcripts lacking stop codons [58]. Interestingly, PARP1 depletion led to an upregulation in the expression of transcripts for protein products involved in the NMD pathway, and a decrease in transcripts of proteins involved in poly-A-RNA binding, showing a clear intersection of PARP1 in RNA biogenesis. Several studies implicate PARP1 in several steps of RNA biogenesis such as RNA metabolism [59], mRNA metabolism, and protein synthesis [3, 60]. Furthermore, splicing factor 3A subunit 1, splicing factor 3B subunit 1, splicing factor 3B subunit 2 [61], and alternative-splicing factor 1/splicing factor 3 [62] are either targets of poly(ADP-ribosyl)ation or bind directly to PARP1. The function of poly (ADP-ribose) binding, the binding to PARPs, and ADP ribosylation of these splicing factors is not well understood.

In these studies, we show that PARP1, a known DNA-binding protein, binds RNA both *in vivo* (Figure 1 and Supplementary Figure S1) and *in vitro* (Figure 7). Our forward competition assays of PARP1 binding to DNA and RNA showed that, in the absence of the Zn1Zn2 domain, PARP1 preferred binding to RNA than to DNA (Figure 8). These results are consistent with our idea of PARP1’s role in co-transcriptional splicing [20], where PARP1 binds to chromatin using the Zn1Zn2 domain, and when that site is used it still has the ability to bind to nascent mRNA through another domain. *Does PARP1 recognize a specific RNA motif?* Previous studies showed that PARP1 binds the DNA motif, AGGCC [63], and/or binds to the vicinity of the DNA motif, GGAAGG [64]. In our analysis, we failed to find an enriched RNA motif for PARP1 binding; we did, however, find that PARP1 binds to RNA sequences enriched in GC-rich sequences (Figure 3 and Supplementary Figure S6). It is tempting to speculate that in binding to these GC-rich sequences PARP1 recognizes a structure formed by these sequences. One such structure is formed by G-quadruplexes, which have also been implicated in splicing regulation. In fact, PARP1 binds G-quadruplexes *in vivo* [65–67]. However, additional studies will be needed to test whether PARP1 RNA targets form structures such as the G-quadruplex.

Our results showing that deletion of the third zinc finger of PARP1 resulted in the lowest affinity of this mutant protein for RNA (Figure 7 and Table 2) support the idea that PARP1 uses its Zn3 to bind RNA *in vitro* [34] or pRNA [17]. The small difference in affinity between PARP1-FL and its truncation mutants could imply that either: (i) all regions contribute to RNA binding or (ii) as yet, there is an undiscovered RNA-binding region of PARP1. These possibilities are not far-fetched since other PARPs lacking of some of the domains of PARP1 bind to RNA. For instance, PARP12 and PARP13 bind RNA through its zinc fingers, whereas PARP14 and PARP10 have possible RRMs present on different protein domains [68]. In addition, PARP7, which lacks these zinc-finger domains, still binds RNA [69]. As of now, it is not clear whether there is an RNA recognition motif on PARP1, although in addition to the zinc-finger 3 domain the WGR domain can also bind RNA [34]. Future studies will be critical to determine the exact RNA recognition motif of PARP1.

In light of PARP1’s *in vivo* binding to RNA, its effect on splicing, and its importance in the regulation of transcript expression of some of the proteins important for NMD and poly-A binding, it is provocative and highly suggestive to hypothesize that PARP1 is a protein involved in genome surveillance. This hypothesis seems plausible if one considers its role in DNA repair; whereas PARP1 does not execute the repair itself, it binds to the site of damage and recruits repair proteins to the site of repair [1]. Furthermore, in transcription regulation, it stalls polymerase elongation [6, 70], thereby possibly allowing proper genome surveillance. Once surveillance is complete, in the absence of any DNA damage, it then PARylates histones, releasing the repression on polymerase elongation [5, 6]. We believe that this is also a likely scenario in splicing. PARP1 by itself does not splice, but binds to specific splice sites [20] (Figure 5), possibly recruiting/activating splice factors to that region. Although recruitment of splice factors has not been shown, PARP1 PARylates and activates splicing factors [62]. In addition, this idea is also further bolstered when one considers its functions at the 3′ ends of mRNA where PARP1 PARylates poly-A binding protein (PAP), thus decreasing the ability of the modified PAP to bind RNA. This PARylation effect has also been shown for several other 3′ processing factors such as PABPN1 and all CPSF subunits [59], pointing to the possibility that PARP1 might be a general regulator of 3′ processing. Lastly, the study of PARP1 under different scenarios has probably led to the idea that it acts in so many functions; however, it is also tempting to speculate that it acts generally as surveillance molecule that ensures genome stability.

Our understanding of the role of PARPs and PAR in transcriptional and post-transcriptional regulation of gene expression through modulation of RNA is still in its early stages. Our studies, however, provide a very useful platform to begin to tease, uncover, and decipher PARP1’s role in the many steps in RNA biogenesis.

## Materials and Methods

### Cell culture

HeLa cells were used for PAR-CLIP experiments. Cells were grown at 37 **°**C in a humidified environment containing 5% CO_2_ and 95% air in Dulbecco’s modified Eagle’s medium (Sigma) containing 1 mM sodium pyruvate, 0.1 mM nonessential amino acids, and supplemented with 10% fetal bovine serum, 100 U ml^−1^ penicillin, and 100 μg/ml streptomycin. For each experiment, ~6×10^8^ cells (~ 60×15 cm cell culture plates) were used.

### PAR-CLIP methodology

Cells were cultured to 80–90% confluency, and then treated overnight with 4-thiouridine to a final concentration of 100 μM added directly to the cell culture medium. Cells were washed with ice-cold phosphate-buffered saline (PBS), the liquid was aspirated and the plates placed over ice and then irradiated with UV light at 365 nm (150 mJ cm^−^^2^). Cells were then scraped off the plates and collected by centrifuging at 2000 r.p.m. for 10 min.

PAR-CLIP was performed as previously described [26] with some modifications. Briefly, 10 ml of packed cell pellet-UV-treated cells were lysed with 3 volumes of 1× NP40 lysis buffer on ice for 5 min. Cells were pelleted by centrifugation at 18 000*g* for 15 min using an Eppendorf 5810R centrifuge 5810R with an A-4-81swing bucket rotor. The supernatant was filtered using a 5 μM syringe filter (Sterile Acrodisc Syringe Filters with Supor Membrane; Ann Arbor, MI 48103 USA) to remove cellular debris. The filtrate was partially treated with RNase T1 (Roche, Pleasanton, CA, USA) to a final concentration of 1 U μl^−1^ for 15 min. The RNase-treated supernatant was then incubated for 2 h with 600 μl of protein A dynabeads (Invitrogen, Thermo fisher Scientific, Waltham, MA,USA) bound to 15 μg of anti-PARP1 antibody (Active Motif, Carlsbad, CA, USA) or control IgG antibody. The beads were washed three times and the immunoprecipitated RNA was digested again with RNase T1 to a final concentration of 63 U μl^−1^ for 15 min. After dephosphorylation, the RNA segments crosslinked to PARP1 were 5′-radiolabeled using γ-^32^P-ATP and T4 polynucleotide kinase (Promega Madison, WI, USA) in one original bead volume. After several washes, each CLIP sample (on the beads) was then treated with 5 U of DNase1 (NEB Ipswich, MA, USA) for every 100 μl of bead volume for 15 min at 37 °C. DNase1 was inactivated by adding 5 mM EDTA and heated at 65 **°**C for 10 min. Samples were then resuspended in SDS-PAGE loading buffer, incubated at 95 °C for 5 min to denature, and the PARP1-RNA crosslinks were release. The samples were then separated on 4–12% NuPAGE gels (Invitrogen) and transferred onto nitrocellulose membranes (1/10th of the sample was used for immunoblotting and the rest of the sample was used for autoradiography). The gel containing 1/10th of the sample and the membrane containing 9/10th of the sample were exposed to a phosphorimager screen overnight and visualized by scanning on a Typhoon FLA 9500. PARP1–RNA complexes were cut from the membrane, treated with proteinase K (Roche), followed by Phenol/Chloroform/IAA extractions and ethanol precipitation. The recovered RNA was used for cDNA library preparation.

For this purpose we used NEBNext Multiplex Small RNA Library Prep Set for Illumina (Set 1). Library preparation including 3′ and 5′ SR Adaptor ligations, reverse transcription, and PCR amplification, which were performed according to the manufacturer’s protocol. To remove adaptor-only ligation products, after every step of the protocol (3′ adapter ligation and 5′ adapter ligation) samples were purified using 15% acrylamide-8M Urea gels. Lastly, after limited PCR amplification PCR products were size-selected on a 3.5% NuSieve (Lonza Walkersville MD, USA) low-melting point agarose gel. Expectant PCR products were eluted using the ‘crush and soak’ method, followed by purification using a Qiagen min-elute PCR column. Samples were then first cloned into the Topo TA vector for pilot analysis and then sequenced using 100 bp paired-end sequencing on an Illumina HiSeq 2500.

### Western blot analysis

Protein samples were resuspended in SDS sample buffer, and then separated on 4–12% NuPAGE gel (Invitrogen, Thermo fisher Scientific, Waltham, MA, USA), transferred onto nitrocellulose membranes, blocked with 5% fat-free milk in PBST, and incubated with primary antibodies for 16 h at 4 °C. After several washes with PBST, the membranes were incubated with secondary antibodies conjugated to alkaline phosphatase for 1 h at room temperature, and a signal was developed with ECL reagents (GE Healthcare, Pittsburg, PA, USA). Images were obtained using a Typhoon 9400.

#### Antibodies.

The following antibodies were used in this study: CHIP-grade PARP1 antibody (Active Motif: 39559).

#### PARP1 knockdown.

The ON-TARGETplus Human PARP1 siRNAs (purchased from GE Healthcare Dharmacon, Dharmacon, Lafayette, CO, USA) and DharmaFECT 1 transfection reagent were used to deliver siRNAs into HeLa cells according to the manufacturer’s protocol. In brief, 2×10^5^ cells/well plated on six-well plates were starved by incubating the cells in 2 ml of antibiotic-free complete medium with 10% serum for 2 h. Then, 50 nM of the ON-TARGETplus Human PARP1 siRNAs were added to serum-free DMEM (180 μl) in one tube, and DharmaFECT 1 (2.4 μl) was added to 197.6 μl of serum-free medium in another tube. The contents of each tube were gently mixed individually for 5 min at room temperature and then combined. This mixture was then incubated at room temperature for an additional 20 min. For control samples 2.4 μl of DharmaFECT 1 was added to 400 μl serum-free medium. Subsequently, an additional 1600 μl serum-free medium was added to each mixture of 400 μl for a final volume of 2 000 μl transfection medium and a final siRNA concentration of 50 mM. The starvation media from the cells were then removed and replaced with the 2000 μl transfection mixture. Cells were further incubated for 1 h at 37 **°**C before 10% serum was added. Cells were then allowed to grow for 24–48 h. This procedure was repeated three times, every 24–48 h. After that, the cells were collected and analyzed for mRNA or PARP1 protein to check the target gene knockdown efficiency. A total of seven independent experiments were performed.

#### Purification of human full-length PARP1 and its truncated mutants.

His-tagged PARP1 expression vectors were a kind gift from the Pascal Laboratory (University of Montreal), and purified as previously described [71]. Briefly, the sequences corresponding to full-length PARP1 (aa 1–1 014), ∆CAT (aa 1–662), ∆Zn1∆Zn2 (aa 216–1 014), ∆Zn3 (truncated aa: 232–374), ∆BRCT (truncated aa: 367–494), and ∆WGR (truncated aa: 518–654) were cloned into pET28 expression vector. Proteins were expressed in One Shot BL21 (DE3) pLysS competent cells (*E. coli*) and purified using three subsequent chromatographic fractionations: (1) a Ni^2+^ affinity column (Ni-NTA agarose, Qiagen Valencia, CA, USA), (2) a heparin column (5-ml HiTrap Heparin HP Column, GE Healthcare), and (3) a gel filtration column (Superdex S200 size exclusion column, GE Healthcare, Pittsburg, PA, USA). Pooled fractions were required to monitor expression, purity and analyze fractions; we used SDS-PAGE (NuPage, 4-12% Bis-Tris, Invitrogen). The desired fractions were then concentrated using an Amicon Ultra spin concentrator with a 10 000 molecular weight cutoff (Millipore, Billerica, MA 01821 USA). Protein concentrations were determined with the Pierce BCA Protein Assay Kit (Thermo Scientific, Thermo fisher Scientific) and by absorbance at 280 nm using the molar extinction coefficients calculated for each PARP1-protein: 1.19×10^5^ M^−1 ^cm^−1 ^(PARP1-FL), 1.14×10^5^ M^−1^ cm^−1^ (ΔBRCT), 9.23×10^4^ M^−1^ cm^−1 ^(ΔWGR), 9.82×10^4^ M^-1^cm^−1^(ΔZn3), 8.82×10^4^ M^−1^ cm^−1^ (ΔZn1ΔZn2), and 8.43×10^4^ M^−1^cm^−1 ^(ΔCAT).

### Electrophoretic mobility-shift assay

Electrophoretic mobility-shift assay (EMSA) analysis was performed according to standard procedures. RNA oligonucleotides (19-mer with the sequence: CGUACGCGGGUUUAAACGA) containing the binding sites for PARP1 were labeled at the 5′ termini with ^32^P. For binding assays, a constant amount (0.05 μM) of labeled RNA probe was preincubated with increasing concentrations of PARP1 protein range 0–2.5 μM in a final volume of 20 μl at room temperature for 30 min in 25 mM Tris (pH 7.5), 75 mM NaCl, 50 mM arginine, 0.1 mM TCEP, and 0.1 μg μl^−1^ bovine serum albumin. The RNA–protein complexes were then analyzed by electrophoresis on native 10% polyacrylamide gels (75:1 acrylamide:bisacrylamide) in Tris-borate-EDTA buffer, followed by autoradiography. Autoradiographic images were captured on a storage phosphor screens (type GP, GE Healthcare, Pittsburg, PA, USA) detected with a Typhoon FLA 9500 and quantitated with Image-Quant TL software (GE Healthcare).

Self-consistent estimates of binding stoichiometry (*n*) and the association constant (*K_n_*) were obtained by the method of Fried and Crothers [72, 73]. For a single binding step in which *n* protein molecules associate with RNA (R is used to represent RNA in the equation) the association constant is *K_n_*= [P*_n_*R] / [R][P]*^n^*_free_. Separating variables and taking logarithms gives:

$$\ln\left(\left[P_{n}R\right]/\left[R\right]\right)=\mathrm{n ln}{\left[P\right]}_{free}+{\mathrm{ln K}}_{n}$$

For these experiments, [R]_total_<<[P]_total_, so [P]_total_ is an acceptable estimate of [P]_free_. Thus, a graph of ln([P*_n_*R]/[R) as a function of ln[P]_free_ has a slope equal to the stoichiometry of the binding step, *n*. The equilibrium constant is most simply estimated at the midpoint, where ln([P*_n_*R]/[R])=0 and lnK*_n_*=−*n* ln[P]_free_. Because the assessed stoichiometries differ for different complexes, we estimated the equilibrium constants for the overall reactions, K*_n_* (M^−*n*^) and the corresponding monomer-equivalent association constants, *K* (M^−1^).

*For competition assays*, equimolar amounts (0.05 μM) of radiolabeled ssRNA and radiolabeled ssDNA of the same nucleotide sequence were mixed in a 20 μl reaction and incubated with increasing concentrations of PARP1-FL or its truncated mutants (0–2.5 μM). The binding reaction was performed as described above. The ratio of binding affinities *K*_DNA_/*K*_RNA_ was determined from the relationship [72, 73]

$$K_{DNA}/K_{RNA}=\left(\left[P_{m}D\right]/\left[D\right]{\left[P\right]}^{m}\right)/\left(\left[P_{n}R\right]/\left[R\right]{\left[P\right]}^{n}\right)$$

Where *m* and *n* are the stoichiometries of the binding of protein to DNA and RNA, correspondingly, calculated from single titration experiments of PARP1 proteins (PARP1-FL and mutants) to DNA and RNA (PARP1–DNA titration is not shown). As PARP1–RNA and PARP1–DNA complexes co-migrate under the electrophoretic conditions of our experiments, [P*_m_*D] and [P*_n_*R] were calculated according to the relationships [P*_m_*D]=([D]_0_–[D]_free_)×*m* and [P*_n_*R]=([R]_0_–[R]_free_)×*n*. [D]_0_ and [R]_0 _are the initial concentrations of DNA and RNA, respectively, and [D]_free _and [R]_free_ are the free concentrations of the competitors at each PARP1 concentration in the initial titration experiment. Because all the components of the equation can either be measured or calculated from our experimental data, *K*_DNA_/*K*_RNA_ for the modest values of [PARP1] was calculated from the plots and the ratio of binding affinities estimated from the linear part of the plot by extrapolation to the [PARP1]=0.

#### RNA markers used in PAR-CLIP experiments.

19-mer: CCGUACGCGGGUUUAAACGA

24-mer: CGUACGCGGAAUAGUUUAAACUGU

#### ssRNA used for gel-shift.

19-mer: UAGGCACCGGCAUCUUGAC

20-mer: CCGUACGCGGGUUUAAACGA

24-mer: CGUACGCGGAAUAGUUUAAACUGU

#### ssDNA used for gel-shift.

19-mer: TAGGCACCGGCATCTTGAC

#### DNA and RNA sequences used for PARP1 activation studies.

dsDNA is the 601 widom sequence: 5′-ctggagaatcccggtgccgaggccgctcaattggtcgtagacagctctagcaccgcttaaacgcacgtacgcgctgtcccccgcgttttaaccgccaaggggattactccctagtctccaggcacgtgtcagatatatacatcctgt-3′

dsRNA was made from *in vitro* transcription of the widom sequence using the MEGAscript T7 Transcritpion kit (AM1334).

#### PARP-1 enzymatic assay.

PARP1 (constant at 1 μM) and 'activators' (DNA or RNA; 1–2 μM) were mixed to a final volume of 20 μl in 50 mM Tris (pH 8), 50 mM NaCl, 10 mM MgCl_2_, and 1 mM DTT and allowed to incubate for 1 h at 30 °C. Twenty microliters of the NAD^+^ stock (1 mM) were added to the above tubes for the final 500 μM [NAD^+^]. Reactions were quenched after 1 min with 5×Laemmli buffer, were immediately boiled for 3 min, and were analyzed by 8% SDS-PAGE. Gels were stained with coomassie. If the protein is active, with NAD^+^ in the presence of 'activators', it makes higher molecular weight smeared band.

#### Bioinformatic analyses.

*CLIP-seq analyses*: Replicate libraries were multiplexed and sequenced on an Illumina Hi-seq 2500 using 100 bp paired-end sequencing. Each library yielded between 0.9 and 97 million unique reads (Supplementary Table S1). Biological replicates were performed to avoid possible confounds in the data sets introduced by Illumina sequencing artifacts; all bioinformatics analyses were performed independently for each CLIP sample and all conclusions were independently validated for all CLIP samples. Since very similar conclusions were obtained, the replicates were combined for the subsequent analyses.

Adapters and primers were trimmed from the sequences using a custom script trimAdapters.pl, which incorporated Trimmomatic v0.33 [74]. Quality control was checked using FastQC [75] v0.11.4. Trimmed reads were then concatenated as single-end reads and aligned to the human hg38 genome assembly. Reads were then aligned to the genome both without a reference transcriptome using bowtie v1.1.1 [76] and with a reference transcriptome using tophat v2.0.13 [77, 78] and the Ensembl v82 gtf [79, 80]. PAR-CLIP peaks were analyzed using PARalyzer (v1.5) [29]. The PARalyzer analysis required several steps. In addition to considering the samples independently, the samples were combined as they theoretically represent technical replicates. The first step to prepare sequences for PARalyzer was to filter the sequences using fastx_collapser v0.0.14, which is part of the FASTX Toolkit [81]. The collapsed sequences were then aligned to the human hg38 reference genome assembly using bowtie with the PARalyzer-suggested parameters. Results from PARalyzer were then parsed into evidence and sequence files using a custom script, which prepares the files for input into the motif detection program cERMIT v1.0.1 [31]. Correlation between replica was performed as follows: normalized read counts for each PAR-CLIP peak were obtained by first dividing the total number of aligned reads in the sample by one million and then dividing the number of reads for each peak by this value. A Pearson correlation was calculated on normalized read counts for intersecting peaks across the samples. Intersecting peaks were those peaks covering the same genomic location by one or more nucleotides.

#### Genomic feature determination.

In order to assign mappings to each of the PAR-CLIP peaks (including intronic, exonic, intergenic, promoter, 5′ UTR, and 3′ UTR), each of these regions was marked on the hg38 assembly of the human genome using Ensembl genes and transcripts [80]. Annotations for each region were obtained using the Biomart [82] tools from Ensembl for the GrCh38 (hg38) assembly. A custom perl script was used to parse out each of these features into chromosome-specific files. Once all files were parsed, all of the regions of each chromosome were assigned to a value. The peaks (in bed format) along with the alignments for the collapsed sequences (in bowtie format) were used to create genome tracks for the UCSC genome browser (see Supplementary Data 1).

#### Motif analysis.

K-mer enrichment motif analysis was carried out calculating 4-mer enrichments by sliding within a 20-nt-long window along PAR-CLIP clusters and using the shuffled (10 000 times) hg38 human protein-coding open reading frames as background sequences.

#### Exon–intron and intron–exon boundary analyses.

Exonic regions on the *Homo sapiens* reference genome assembly hg38 were retrieved from the Ensembl database [83]. The exonic regions were filtered to include only exons on protein-coding transcripts. In addition, duplicated exon start and end locations across transcripts for the same gene were removed from the analysis. Specifically, for the analysis of exon to intron boundaries, duplicated exon end locations on the forward DNA strand and duplicated exon start locations on the reverse DNA strand were removed. For the analysis of intron to exon boundaries, duplicated exon start locations on the forward DNA strand and duplicated exon end locations on the reverse DNA strand were removed. This resulted in 281 967 unique exon–intron locations and 278 090 unique intron–exon locations. A custom C++ program was created to count the number of PAR-CLIP sequences covering each base for a distance of 100 bases in the exon and 300 bases in the intron from all exon boundaries. Two complementary methods were used to establish the number of PAR-CLIP sequences expected to cover a genomic region at random. One method utilized the Shuffle tool in the Bedtools package [84] to randomly position exon starting and ending locations while preserving the number and size of exonic regions on individual chromosomes. The second method utilized the Random tool in the Bedtools package to obtain 150 000 random sequences of 5 000 bases in length, the maximum length of the majority of intronic regions [85]. With this method, the starting location for each region represented an exon to intron boundary, and the ending location represented an intron to exon boundary. Half of the sequences were used in the analysis of exon to intron boundaries with the other half used for the analysis of intron to exon boundaries. The C++ program mentioned above was used to count PAR-CLIP sequences covering each base for 100 bases into a simulated exonic region and 300 bases into a simulated intronic region.

#### RNA-seq analysis after PARP1 knockdown.

RNA-seq libraries were constructed using the TruSeq stranded mRNA LT Sample preparation Kit with poly-A enrichment according to the manufacturer's instruction. The libraries corresponded to the three control samples (cells treated with non-targeting, siRNA, Dharmacon Inc.) and three PARP1 knockdown (Dharmacon Inc). These libraries were multiplexed and sequenced on the Illumina NextSeq 500 using the NextSeq 500/550 2×75 cycle High Output Kit v2 (Cat# FC-404-2002).

Differential gene expression and alternative splicing analysis. RNA-seq reads were mapped to the hg38 reference genome assembly using tophat2 (version 2.0.13) [78], generating alignment files in bam format. PARP1 regulated differentially expressed genes were detected using the tuxedo suite of programs including cufflinks-cuffdiff2 (version 2.2.1). Differentially expressed genes were considered significant with *P*-value≤0.05 and |FC| ≥1.

We next identified PARP1-regulated differential alternative splicing events corresponding to five major types of alternative splicing event patterns by rMATS (v3.2.5) [86]. For each alternative splicing event, both the reads mapped to the exon–exon junction and the reads mapped to the exon body were used as rMATS input. Putative PARP1-regulated AS events were identified as those with significant difference in inclusion levels (|ΔPSI|≥5%) between knockdown and control at an false discovery rate (FDR)<5%.

#### rMAPS.

In order to determine the binding patterns of PARP within significantly detected skipped exon events, the PAR-CLIP peaks and all detected skipped/retained exon events were used as input into the rMAPS [33] server. The rMAPS server differentiates between significant skipping and inclusion events, and determines differential binding associated with each type of event in comparison to background introns and exons.

#### Data deposition.

RNA-seq data are deposited in GEO (GSE91051) along with the PAR-CLIP data (GSE95360). The processed files for the PARCLIP peaks, differential gene expression, and alternative splicing analysis are provided as Supplementary Data 1. Visualization tracks for PARCLIP and RNASeq data are provided as a track hub on the UCSC Genome Browser (http://bit.ly/2l7f5OY).

## Figures and Tables

**Table 1:** Length of PARP1-PAR-CLIP reads

k-mer	Number of reads
16-mer	2 142
17-mer	3 897
18-mer	5 271
19-mer	6 894
20-mer	8 935
21-mer	5 883
22-mer	3 535
23-mer	1 888

Abbreviations: PAR–CLIP, photoactivatable-ribonucleoside-enhanced crosslinking and immunoprecipitation; PARP1, Poly (ADP-ribose) polymerase 1.

**Table 2:** Stoichiometries (*n*) and association constants (*K*_assoc_) for PARP1-FL and truncation mutants to 19-mer ssRNA

Protein type	*K*_assoc _(M^−1^)	Stoichiometry (*n*)
PARP1-FL	(1.95±0.08)×10^6^	1.75±0.20
PARP1-∆WGR	(2.91±0.15)×10^6^	2.33±0.18
PARP1-∆CAT	(1.05±0.07)×10^6^	3.31±0.32
PARP1-∆BRCT	(0.97±0.06)×10^6^	1.17±0.10
PARP1-∆Zn1∆Zn2	(0.86±0.04)×10^6^	1.26±0.11
PARP1-∆Zn3	(0.78±0.03)×10^6^	1.19±0.08

**Table 3:** Relative binding affinities from competition EMSA experiments for PARP1-FL and truncation mutants to 19-mer ssDNA compared to 19-mer ssRNA

Protein type	*K*_DNA_/*K*_RNA_	*K*_RNA_/*K*_DNA_
PARP1-FL	25.3±3.0	0.001±0.02
PARP1-∆WGR	26.5±2.4	0.024±0.011
PARP1-∆CAT	15.7±3.9	0.046±0.021
PARP1-∆BRCT	42.3±1.2	0.001±0.016
PARP1-∆Zn1∆Zn2	0.015 3±0.021	7.2±0.7
PARP1-∆Zn3	23.1±5.2	0.032±0.017
