# Supplementary material for: Transcriptome-wide identification of the RNA-binding landscape of the chromatin-associated protein PARP1 reveals functions in RNA biogenesis
Source: Cell Discov. 2017 Nov 28;3:17043–. doi: 10.1038/celldisc.2017.43 (PMC5787697; doi:10.1038/celldisc.2017.43)
Supplement: Supplementary Information [file celldisc201743-s1.pdf]

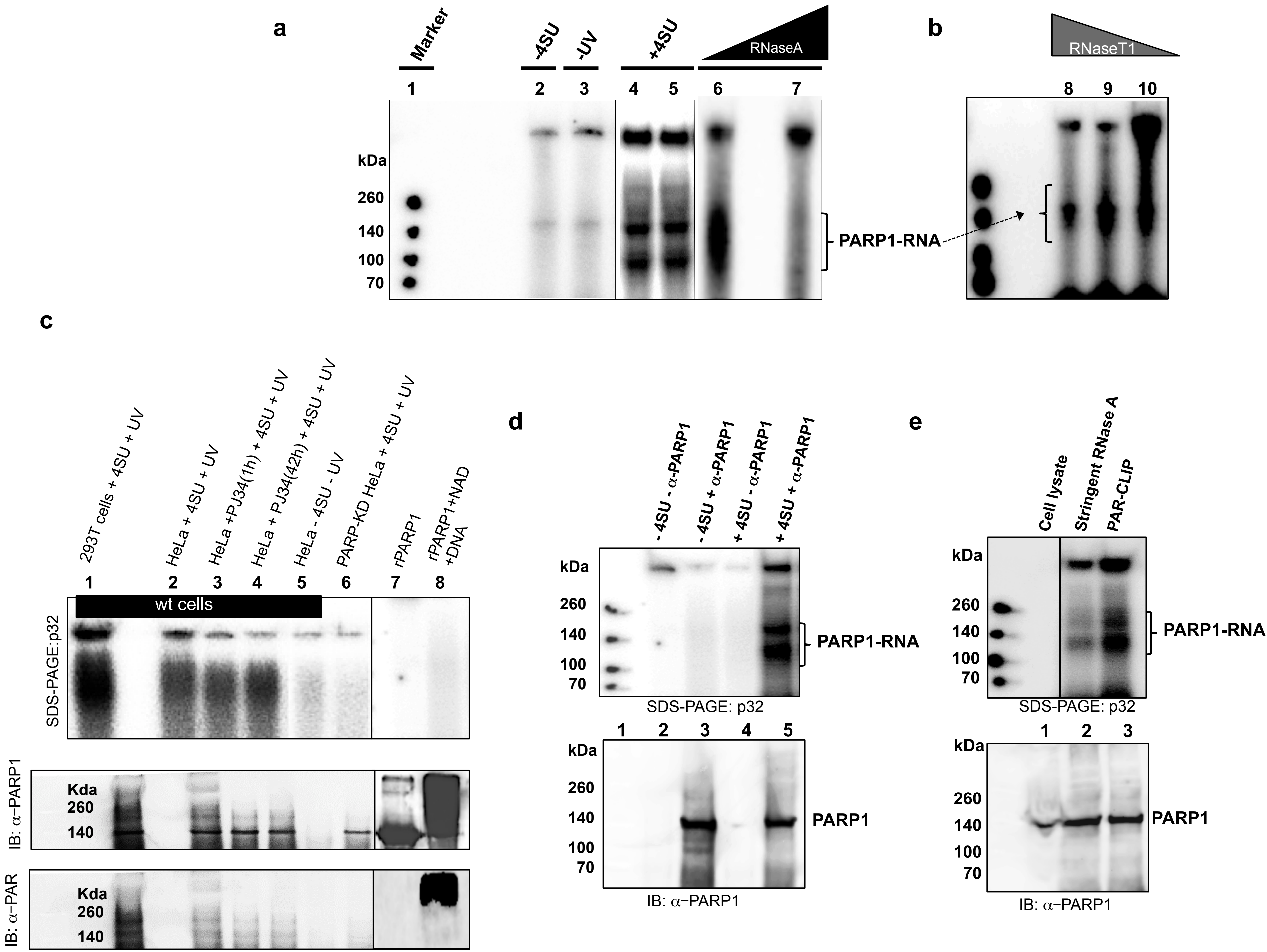
a
Marker
-4SU
-UV
+4SU
RNaseA
1 2 3 4 5 6 7
kDa
260
140
100
70
PARP1-RNA
b
RNaseT1
8 9 10
c
293T cells + 4SU + UV
HeLa + 4SU + UV
HeLa +PJ34(1h) + 4SU + UV
HeLa + PJ34(42h) + 4SU + UV
HeLa - 4SU - UV
PARP-KD HeLa + 4SU + UV
rPARP1
rPARP1+NAD +DNA
1 2 3 4 5 6 7 8
wt cells
SDS-PAGE:p32
IB: α−PARP1
Kda
260
140
IB: α−PAR
Kda
260
140
d
- 4SU - α-PARP1
- 4SU + α-PARP1
+ 4SU - α-PARP1
+ 4SU + α-PARP1
kDa
260
140
100
70
PARP1-RNA
SDS-PAGE: p32
1 2 3 4 5
kDa
260
140
100
70
PARP1
IB: α−PARP1
e
Cell lysate
Stringent RNase A
PAR-CLIP
kDa
260
140
100
70
PARP1-RNA
SDS-PAGE: p32
1 2 3
kDa
260
140
100
70
PARP1
IB: α−PARP1

Supplemental Figure S1: Controls for PAR-CLIP experiments: **a.** In cells not treated with thiouridine, no significant amount of labeled RNA was observed (lane 2). Even when cells were treated thiouridine and not UV crosslinked, very little labeled RNA was observed (lane 3). In the presence of thiouridine and UV-crosslinking at 340nm, we observed a significant amount of PARP1-bound RNA (lanes 4 and 5). Further treatment of this sample with increasing concentration of RNAse A (lanes 5 and 6) resulted in the elimination of the PARP1-RNA band, indicating that this band is indeed RNA. **b.** Control PAR-CLIP experiments, showing digest with increase concentrations of RNAse T1 (lowest to highest RNaseT1 concentration – Lane 1 to Lane 3), reduced the PARP1-RNA smear to bands corresponding to PARP1 size (Lane 3). **c.** PAR-CLIP experiments in 293T cells and in HeLA cells show that PARP1-RNA binding is not cell-type specific. Furthermore, treatment of cells for 1hr (Lane 3) or 24hours (Lane 4) with PJ34 (PARylation inhibitor) did not eliminate the PARP1-RNA binding, showing that this observation is not due to PARylated PARP1. Lastly, knockdown of PARP1 resulted in the elimination of the smear (band)- Lane 6. Top image shows PARP1-RNA imaged with typhoon, and then the same membrane was probed with PARP1 antibody (middle blot) and with PAR antibody (bottom blot), showing that the ~140KDa band is indeed PARP1 and not PARylated PARP1. Recombinant PARP1 (rPARP1 - lane 7) also ran ~140KDa as estimated by the protein ladder used in our experiments. rPARP1 was treated with DNA and NAD to test for PARylation (lane 8), which confirmed that the band at ~140KDa is PARP1 and not PARylated PARP1 (as tested with PARP1 and PAR antibodies respectively). **d.** Control PAR-CLIP experiments were carried out with and without thiouridine or PARP1 antibody. Top part of the gel shows membrane and observation of labeled RNA only when both thiouridine and PARP1 antibody were used in the experiments. Lower part of the gel shows, western blot analysis of PARP1-proteins, further confirming that only in the presence of thiouridine and PARP1 antibody do we see the protein bound to RNA. **e.** Stringent RNaseA digest depleted the PARP1 band indicating that PARP1 is in a complex with RNA and DNA. Lower part of the figure shows western blot analysis of the same membrane showing that PARP1 was present in the complex. *For all experiments we used Pre-Stained markers from ThermoFisher Scientific®, which gives an estimation of the size of the proteins and not absolute sizes.

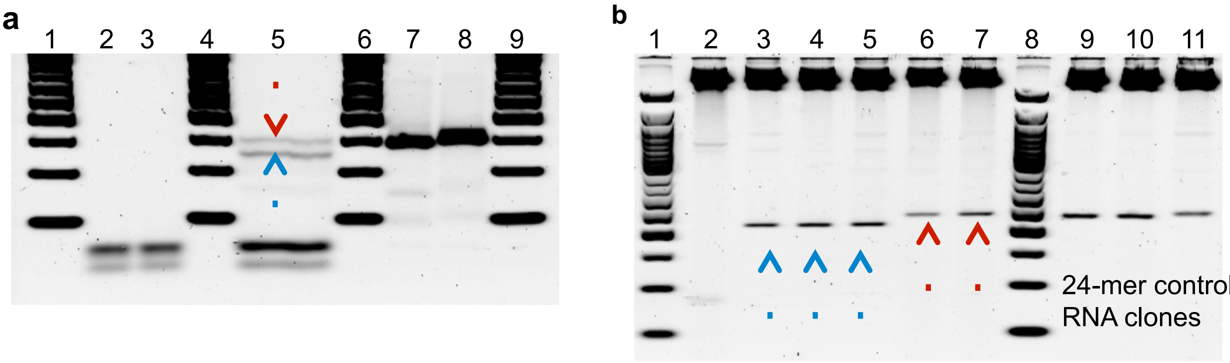

c

<u>Examples of sequences identified through sanger-sequencing</u>

1. CAAATGCCCTTCCC
2. CCCCTCCGCCGCCCGCC
3. ATCCACCGTGCGCCCTT
4. CGCCCGTCCCTTCGGATCG
5. TTTCCCAGCTGATTCTGCTTG
6. CCGACGATGTTCTACAGTCCGA
7. ATCGATGCCAGAGCCAAGAGA
8. CGGACTGTAGAACTCGATCTCGG
9. GTTATGAGTACGACCGGATATGA
10. CAGTCCGCCCCGCCCCCCGACCC
11. ACAACCCTCAACCATATGTAGTCCAAGCA
12. CCGATACTAATGAACCGTGAGGCTTAACC

Supplementary Figure S2. Identification of the PARP1-bound RNAs. PCR amplification of ligated PARP1-bound RNAs and pilot analyses to determine sequences bound by PARP1. **a.** PCR amplification of the adapter-ligated products. Lanes 2 and 3 are control experiments of adapter only PCR amplification. Lane 5 shows the amplification of PARP1-bound products – the band indicated by the red arrow is the correct amplified product while the blue arrow indicates non-specific primer-dimer products. Lanes 7 and 8, indicate products obtained from the control 19-mer RNA marker and 24-mer RNA markers, respectively, subjected to every round of experiment performed on the PARP1-bound RNAs. **b**. Fragments from supplementary Figure S2**a** were cloned into PUC19 vector and the plasmid DNA digested with HindIII and EcoR1 to release ligated products. Lanes 3 – 5 show products obtained from the blue arrow (Supplementary Figure S2a) again confirming their length and composition, while red arrows are the correct product of PARP1 bound products. Lanes 9 – 11 are from the products for 24-mer cloned and digested with same restriction enzymes. **c**. Examples of some of the PARP1-bound sequences from Sanger - sequencing.

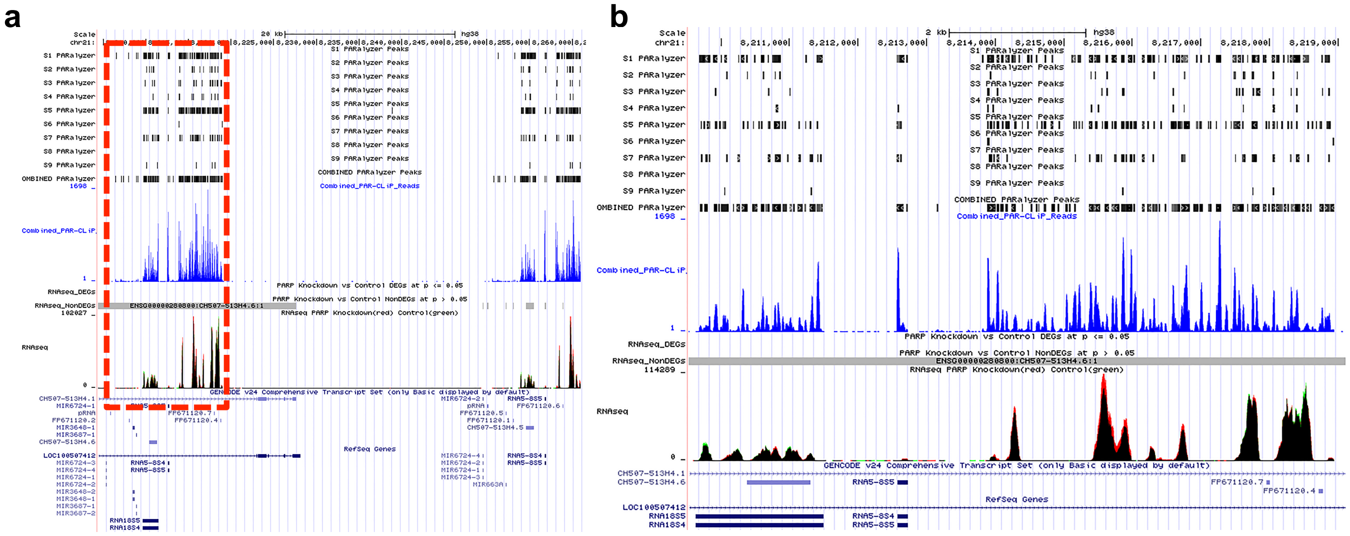

Supplementary Figure S3. Genome browser tracks a. Visualization of individual biological replicates of PARP1-PAR-CLIP peaks for an ~64kb region of hg38 chromosome 21 (positions 8,204,590-8,268,402). **b**. 10kb inset for region highlighted with dotted red line. The vertical black bars in the top panel indicate the location of detected peaks within each of the biological replicates, illustrating the high degree of similarity. The blue profiles in the middle panel indicate the combined position-based read counts for PAR-CLIP sequencing results. The profiles that are red, green and black in the third panel represent the normalized RPKM for the PARP1-depleted RNA-seq.

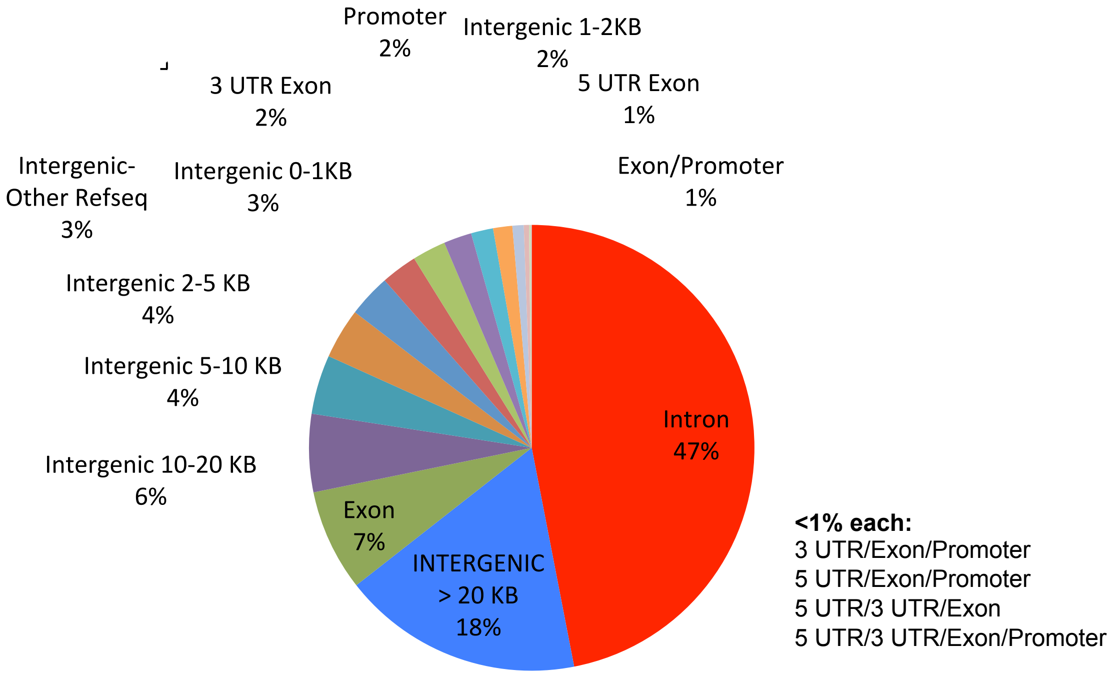

Supplementary Figure S4: The detailed binding sites of PARP1 PAR-CLIP reads identified within different genomic regulatory regions. A pie chart depicting the proportion of PARP1-CLIP-tags within different intergenic as defined in relation to known reference genomes, close to TSSs and intergenic regions, introns, promoters, and UTRs. This analysis showed that ~18% can be defined as true intergenic regions.

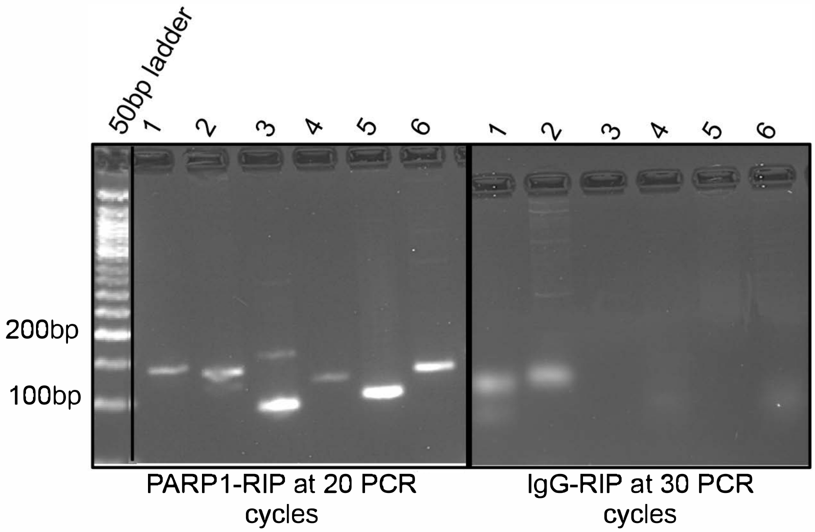

Supplementary Figure S5: Confirmation by formaldehyde-crosslinked RIP of PARP1-RNA binding. Confirmation of PARP1-RNA binding to target sites identified from PAR-CLIP-seq. PCR cycles were done at 20 cycles to show enrichment of target sites while samples from IgG

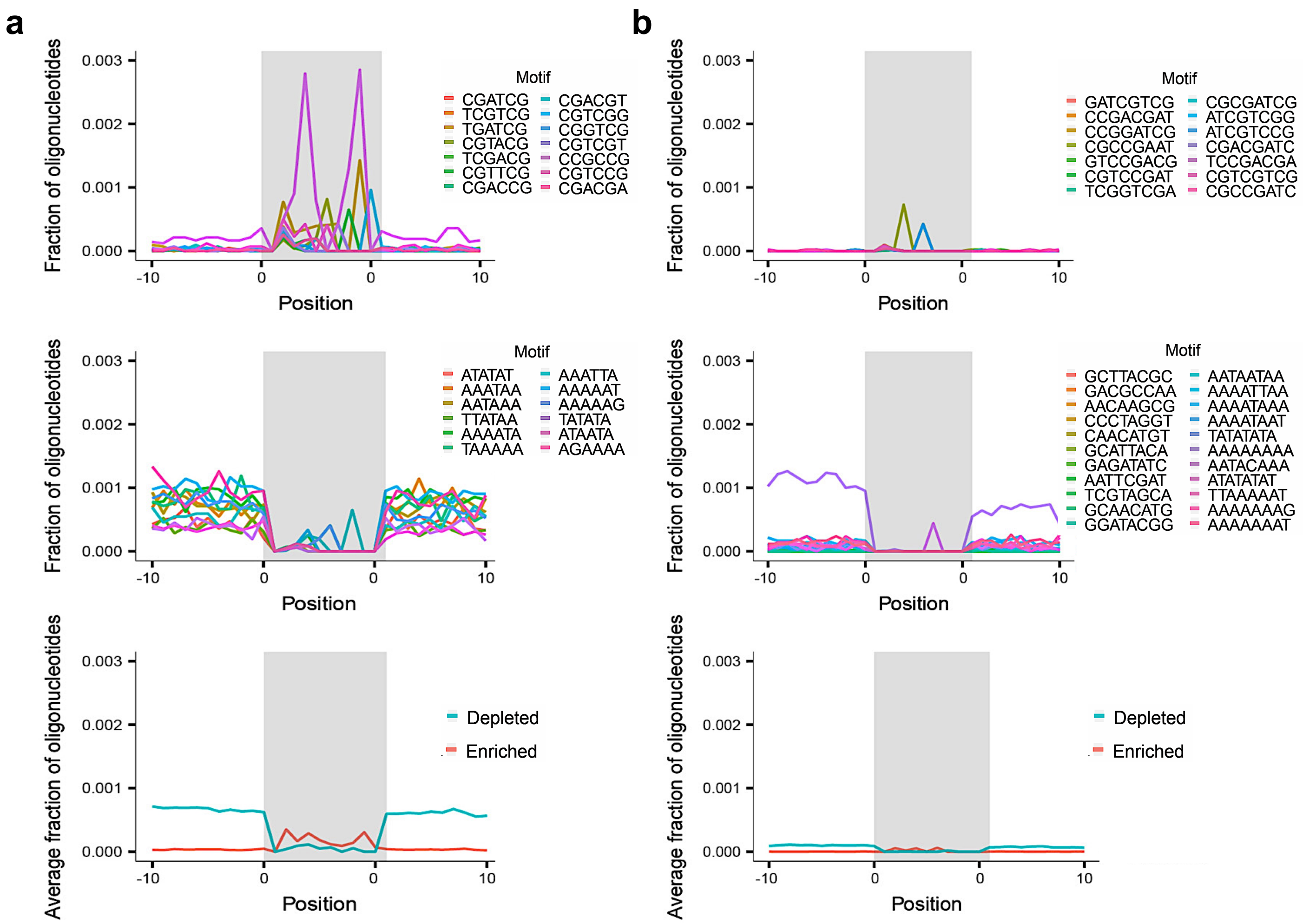

Supplementary Figure S6. 6-mer and 8-mer enrichment analysis of PARP1-PAR-CLIP reads). a. (Upper Panel): Enriched 6-mer motifs found within percentile regions of the PARP1 binding site (gray) along with their enrichment scores in the flanking upstream and downstream regions**. (Middle Panel):** Depleted 6-mer motifs within the PARP1 biding site (gray) along with their enrichment scores in the flanking upstream and downstream regions. **(Lower Panel):** Average enrichment score for enriched and depleted motifs from Upper Panel and Middle Panel, respectively. **b. (Upper Panel):** Enriched 8-mer motifs. **(Middle Panel):** Depleted 8-mer motifs **(Lower Panel).** Average enrichment score for enriched and depleted 8-mer motifs. The enrichment scores for the upper panels are determined by the log2-odds score of the frequency of the motif in the region versus the frequency of the motif in the genome.

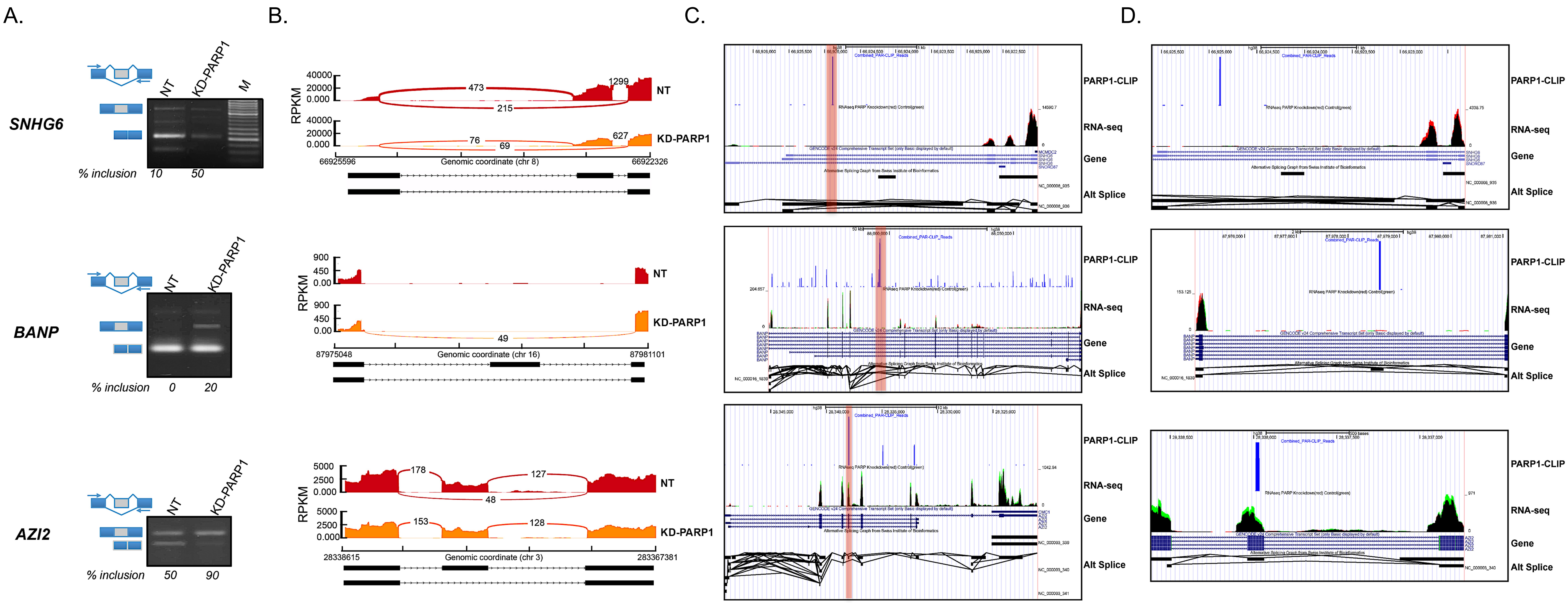

A.
B.
C.
D.
SNHG6
BANP
AZI2
NT
KD-PARP1
M
% inclusion 10 50
% inclusion 0 20
% inclusion 50 90
RPKM
473
1299
215
76
627
69
66925596
Genomic coordinate (chr 8)
66922326
Genomic coordinate (chr 16)
87975048
87981101
49
178
127
48
153
128
28338615
Genomic coordinate (chr 3)
283367381
PARP1-CLIP
RNA-seq
Gene
Alt Splice

Supplementary Figure S7. Altered splicing of transcripts due to PARP1 depletion. a. Validation of splicing defects due to PARP1 depletion by RT-PCR analysis. Arrows indicate the location of the primers used and the percent inclusion of specific exons. Products were cloned into PUC19 for confirmation of the correct products. **b**. Sashimi plot illustrating Tophat-mapped RNA sequencing reads and exon junctions from wt and PARP1-KD cells. Annotated below each Sashimi plot is the gene annotation and the exons. **c**. UCSC browser showing the location of PARP1 bound RNAs (PAR-CLIP) relative to the transcripts (RNA-seq) relative to the alternative spliced products (Alt Splice). Red boxes indicated the regions probed by sashimi plots as well as RT-PCR. In relationship to splicing at the sample genes, PARP1-RNA peaks occur at or just before the alternatively spliced exons. Red highlighted region is the region mapped by Sashimi plots in B. **d**. Zoomed region of highlighted region in **c**.

a

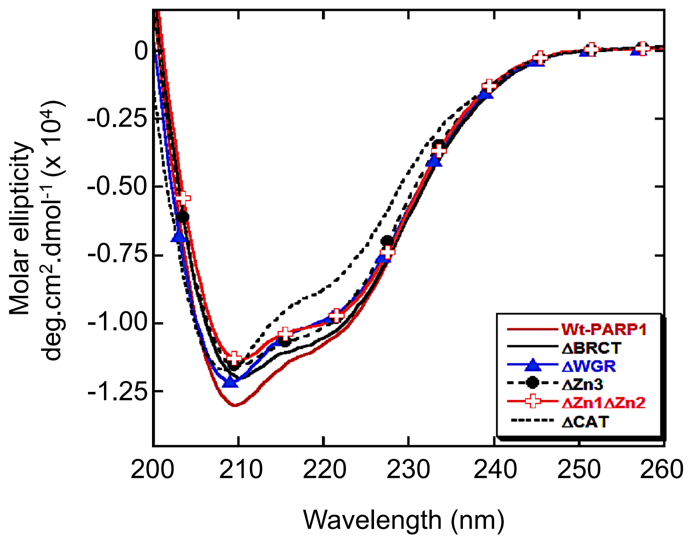
Molar ellipticity
deg.cm2.dmol-1 (x 104)
0
-0.25
-0.50
-0.75
-1.00
-1.25
200
210
220
230
240
250
260
Wavelength (nm)
Wt-PARP1
ΔBRCT
ΔWGR
ΔZn3
ΔZn1ΔZn2
ΔCAT

b

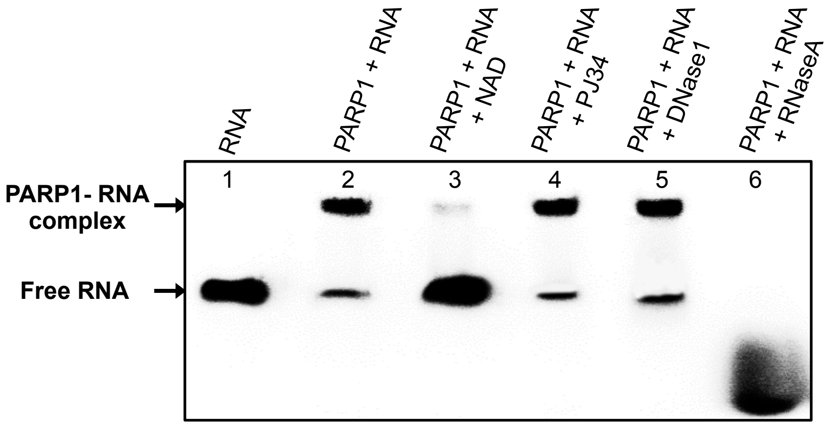
RNA
PARP1 + RNA
PARP1 + RNA + NAD
PARP1 + RNA + PJ34
PARP1 + RNA + DNase1
PARP1 + RNA + RNaseA
1
2
3
4
5
6
PARP1- RNA complex
Free RNA

Supplementary Figure S8: Conformational analyses of PARP1 and identification of the binding species. a. Circular dichroism analysis of the PARP1-FL and truncation mutants. **b**. Enzymatic analyses of PARP1-RNA binding species. PARP1-bound RNA shifted on gelshift assays (Lane 1). PARylated PARP1 (PARP1 treated with NAD+ before incubating with RNA) does not bind RNA (Lane 3), while treatment with PJ34 (PARylation inhibitor) had no significant effect on the PARP1-RNA binding profile (Lane 4). Treatment with DNase 1 (Lane 5) also did not change the binding profile of PARP1-RNA binding, while RNaseA (Lane 6), digested the RNAs, indicating that RNA is the nucleotide species bound by PARP1 in these experiments.

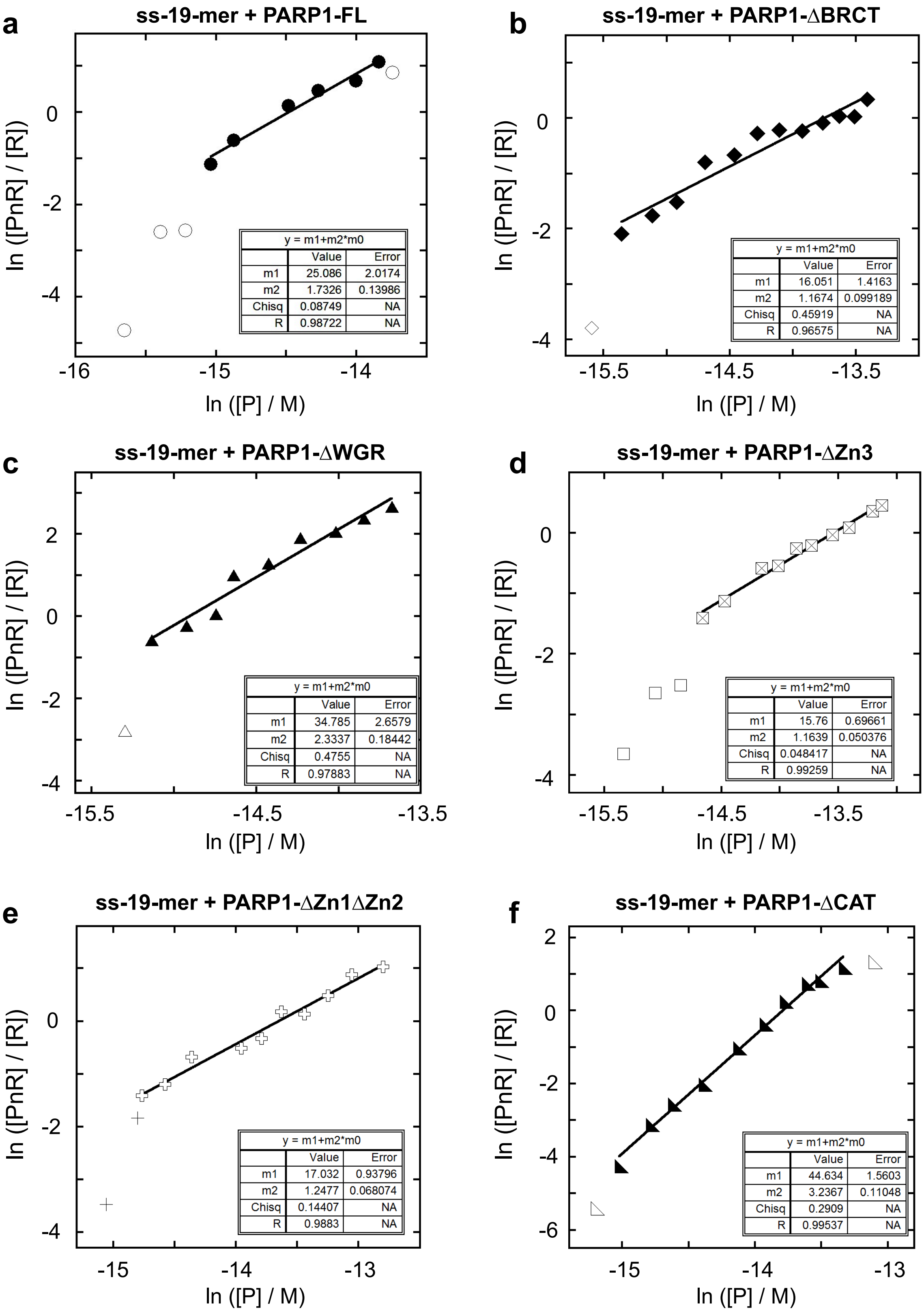
a
ss-19-mer + PARP1-FL
ln ([PnR] / [R])
ln ([P] / M)
y = m1+m2*m0
Value Error
m1 25.086 2.0174
m2 1.7326 0.13986
Chisq 0.08749 NA
R 0.98722 NA
b
ss-19-mer + PARP1-ΔBRCT
ln ([PnR] / [R])
ln ([P] / M)
y = m1+m2*m0
Value Error
m1 16.051 1.4163
m2 1.1674 0.099189
Chisq 0.45919 NA
R 0.96575 NA
c
ss-19-mer + PARP1-ΔWGR
ln ([PnR] / [R])
ln ([P] / M)
y = m1+m2*m0
Value Error
m1 34.785 2.6579
m2 2.3337 0.18442
Chisq 0.4755 NA
R 0.97883 NA
d
ss-19-mer + PARP1-ΔZn3
ln ([PnR] / [R])
ln ([P] / M)
y = m1+m2*m0
Value Error
m1 15.76 0.69661
m2 1.1639 0.050376
Chisq 0.048417 NA
R 0.99259 NA
e
ss-19-mer + PARP1-ΔZn1ΔZn2
ln ([PnR] / [R])
ln ([P] / M)
y = m1+m2*m0
Value Error
m1 17.032 0.93796
m2 1.2477 0.068074
Chisq 0.14407 NA
R 0.9883 NA
f
ss-19-mer + PARP1-ΔCAT
ln ([PnR] / [R])
ln ([P] / M)
y = m1+m2*m0
Value Error
m1 44.634 1.5603
m2 3.2367 0.11048
Chisq 0.2909 NA
R 0.99537 NA

Supplementary Figure S9: Associated binding isotherm analysis for each of the PARP1 proteins to single-stranded 19-mer RNA. Associated binding isotherm analysis. The slope of each isotherm is a measure of stoichiometry for the individual PARP1 protein binding to the RNA: **a**. PARP1-FL, **b**. ΔBRCT, **c**. ΔWGR, **d**. ΔZn3, **e**. ΔZn1ΔZn2, **f**. ΔCAT. Values of n and K are summarized in Table 2 and Supplementary Table 5.

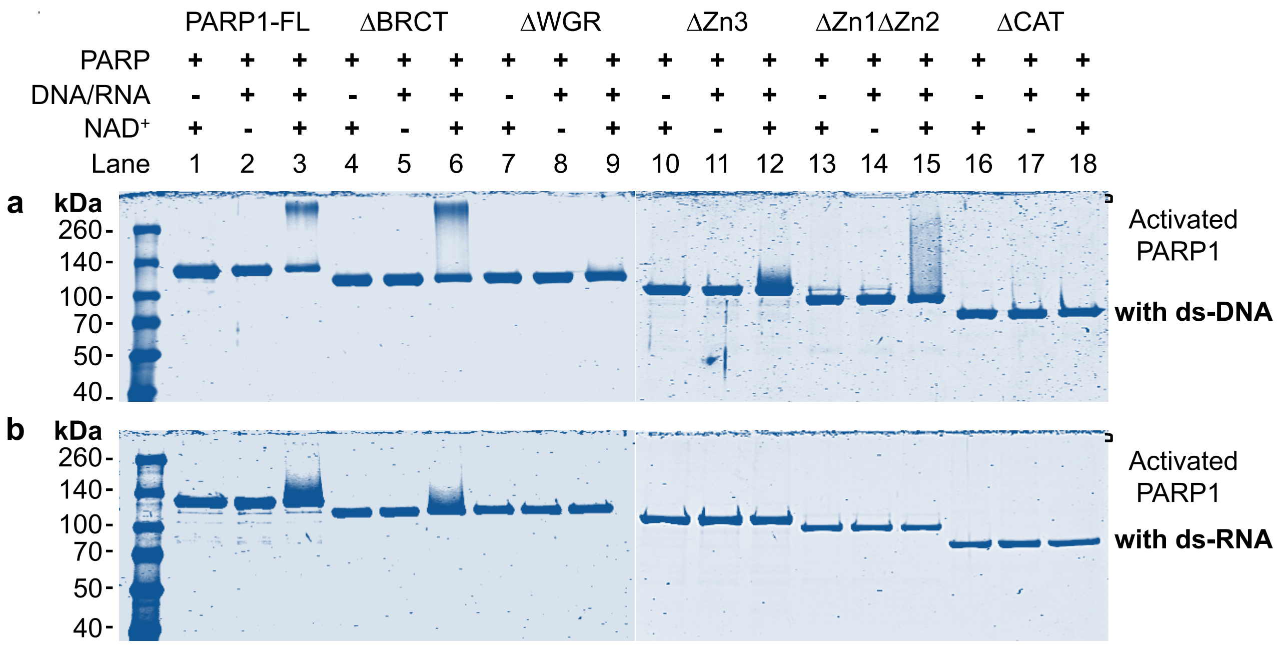

Supplementary Figure 10. RNA activates PARP1 similar to DNA, except to a lower extent. PARylation activation assays were performed in the presence of NAD^+ and DNA (**Supplementary Figure S10a**) or RNA (**Supplementary Figure S10b**) using PARP1-FL and the various truncation mutants as indicated above the gels. Reactions in lanes 1, 4, 7, 10, 13 and 16 have respective proteins with NAD+; lanes 2, 5, 8, 11, 14 and 17 have proteins incubated with either DNA (**Supplementary Figure S10 a**) or RNA (**Supplementary Figure S10b**); and lanes 3, 6, 9, 12, 15 and 18 represent incubation of either DNA or RNA with proteins and NAD^+ and resolved on by SDS-PAGE. Smear above the PARP1 bands indicate PARP1 PARylation and activation. PARP1 activation by DNA or RNA is seen as follows – PARP1-FL > ΔBRCT > ΔZn1ΔZn2 >ΔZn3. No activation was seen as expected with ΔWGR and ΔCAT (Lanes 9 and 18)

Supplementary Table S1: Summary of the PAR-CLIP data sets and analysis from the biological replicates from PARP1-PAR-CLIP sequencing.

Sample Name	Raw Reads	Raw Bases	Paired Reads After Trimming	Paired Bases After Trimming	Single Reads After Trimming	Single Bases After Trimming	Overall Mapping Rate	Replica Pearson Correlation $p < 0.05$
S1	97,282,512	19,456,502,400	39,484,058	1,676,666,672	12,498,768	231,046,589	46.9%	1
S2	37,189,800	7,437,960,000	3,585,764	141,158,177	1,423,784	25,396,958	52.6%	0.88
S3	18,872,232	3,774,446,400	2,041,922	81,634,711	828,137	14,885,583	52.9%	0.79
S4	2,094,387	418,877,400	149,084	6,477,546	101,461	2,185,216	48.2%	0.52
S5	13,783,503	2,756,700,600	4,000,443	165,381,745	2,527,501	49,720,237	53.4%	0.69
S6	13,309,898	2,661,979,600	2,808,984	119,010,345	2,614,978	54,268,707	45.6%	0.8
S7	441,374	88,274,800	64,241	3,095,732	123,122	3,628,587	37.9%	0.5

Supplementary Table S2: PARP1-CLIP tags mapping to 'intergenic' regions that are close to either start or stop of reference genes or to genes in other referenced genomes

**Genomic regions**	**Number of intergenic PARP1-PAR-CLiP-tags mapping to different regions**
Other RefSeq genomes	1360
0 – 1 kb	1145
1 – 2 kb	701
2 – 5 kb	1600
5 – 10 kb	1873
10 – 20 kb	2468
Intergenic > 20 kb	7627

Supplementary Table S3: RNA types mapped by PARP1-PAR-CLIP reads

RNA type	Total number of PARP1-PAR-CLIP peaks	% of the RNA type mapped to total mapped PARP1-PAR-CLIP peaks	Total number of known RNAs	% of PARP1 PAR-CLIP peaks mapped to known RNAs
mRNA	22,142	88%		
lincRNAs	2,870	11%	121,630	13%
miRNA	124	5%	939	13%
snoRNA	88	4%	402	22%
Total	25224	100%		

Supplementary Table S4: Pearson correlation of biological replica of RNA-seq experiments for NT and PARP1-knockdown cells

	**wt-rep1**	**wt – rep 2**	**knockdown – rep1**	**knockdown – rep2**
**wt-rep1**	1.000	0.983	-	-
**wt-rep2**	0.983	1.000	-	-
**knockdown – rep2**	-	-	1.000	0.982
**knockdown – rep2**	-	-	0.982	1.000

Supplementary Table S5. Genes differentially expressed in PARP1 knockdown conditions at $p \leq 0.05$, $q \leq 1$, $|FC| \geq 1$.

**Upregulated DEGs (217)**				
ACP5	H2AFX	PLA2G16	DPM3	ENSG00000073169
ALDOA	HES4	PLAU	DPP7	ENSG00000202343
APRT	HIF3A	PLPP2	DRAIC	ENSG00000214870
ARID5A	HIST1H2AE	PODNL1	EBI3	ENSG00000217801
ATP5D	HIST1H2BE	PPP1R14B	EGFL7	ENSG00000222881
ATP5G1P4	HIST1H2BM	PRELID1P1	EIF4EBP1	ENSG00000224208
AURKAIP1	HIST1H3B	PRRX2	EPN1	ENSG00000225218
B3GAT3	HMGN1P9	PRSS56	ETFB	ENSG00000227586
BBC3	HPN	PRSS8	FCGRT	ENSG00000230202
BCAT2	HSPB1	RHPN1	FDXR	ENSG00000236773
BLVRB	ICA1L	RN7SL76P	FKBP2	ENSG00000237846
C12orf57	ICAM5	RNU2-63P	FKBP8	ENSG00000249072
C9orf142	IGFBP6	RNU5B-2P	FSCN1	ENSG00000254780
CCDC124	IL1B	RNU6-450P	FXYD6P2	ENSG00000255301
CCDC167	INAFM1	RNY3P16	GDF15	ENSG00000258798
CCDC85B	ISG15:	RPL13P12	GDPD5	ENSG00000258884
CCDC88B	ISOC2	RPL18A	GIPR	ENSG00000259015
CD70	JOSD2	RPL36	GLIS1	ENSG00000259884
CDA	JUP	RPL8	GOLGA7B	ENSG00000260018
CDT1	KCNU1	RPLP1	GPX3	ENSG00000261431
CHAC1	KLF16	RPLP2	GPX4	ENSG00000267605
CHCHD10	KLK2	RPS15	H1FX	ENSG00000269103
CHMP2A	KRT13	RPS19	H2AFJ	ENSG00000271745
CITED4	KRT17	RPS19BP1	MFSD10	ENSG00000272473
CKB	LAGE3	RPS5	MFSD3	ENSG00000272562
CLIC3	LAMTOR4	RSAD2	MIDN	ENSG00000273001

**Upregulated DEGs (217)**				
CLPP	LDLRAD4	SDF2L1	MIF-AS1	ENSG00000273321
CNFN	LGALS1	SERPINC1	MMP15	ENSG00000273416
COL11A2	LIMK1	SFN	MPND	ENSG00000273712
COMTD1	LINC01272	SGCA	MRPL41	ENSG00000273727
CRIP1	LRRC32	SIGIRR	MT1XP1	ENSG00000274012
CRIP2	LRRC36:	SIVA1	MT2A	ENSG00000274213
CST3	MAPK11	SLC25A41	NALT1	ENSG00000274341
CTAG2	MAPK8IP1	SLC6A10P:	NDUFA11	ENSG00000278642
CUEDC2	MED16	SLC8A1-AS1	NDUFA13	ENSG00000279560
CYBA	MEIS3	SNCG	NDUFB7	ENSG00000280176
DCXR	MESP1	SNHG19	NDUFS6	ENSG00000280325
NME3	SNHG9	TNFRSF14	ENSG00000281117	XAB2
NRTN	SRRM3	TP53I11	NXPH4	YPEL3
NUDT1	SSNA1	TSC22D4	TLX3	TMUB1
NUPR1	STK32C	TSPO	TULP2	TNFRSF12A
PACSIN3	TMEM158	UBALD2	PALM	UBTD1
PAEP	TMEM184A	UBBP4	PKN1	WDR49
WNT6	ZNF165			

**Downregulated DEGs (81)**				
ALDH1A2	MMP2	RNU6ATAC	GALNT1	ENSG00000228873
APOBEC3G	NACAP1	RNVU1-1	GATSL2	ENSG00000232063
ATP5EP2	NEK10	RYR3	HLTF	ENSG00000232372
BMS1P1	NEK7	SCFD2	HMMR	ENSG00000237611
BRD9P2	NMD3	SEPT14P19	ITGB1	ENSG00000238035
CCDC88A	NOC3L	SGMS2	KAT2B	ENSG00000254731

COG6	NPLOC4	SNORA67	LGALS4	ENSG00000260366
CTGLF9P	**PARP1**	TCEB1P2	LINC01554	ENSG00000260766
DCLRE1A	PDE1A	TNFRSF13C	LTN1	ENSG00000270808
DCLRE1CP1	PEAK1	TRPC1	PLA2G4A	ENSG00000271973
EXTL2	PFN1P2	TTC37	PLEKHM3	ENSG00000273682
FAM27D1	PKD2L1	ZC3HAV1L	PLXNA2	ENSG00000273816
POU2F2	ZMYM1	ENSG00000224100	POMK	ENSG00000274422
PRKAR2A	ZNF346	ENSG00000224631	ENSG00000278077	ENSG00000275675
RAPGEF6	ZNF93	ENSG00000228463	ENSG00000278099	ENSG00000276412
RNU6-945P	ENSG00000221044	ENSG00000228527	ENSG00000280217	ENSG00000276997
				ENSG00000280331

Supplementary Table S6: Stoichiometry (n) and association constants (K_{assoc}) for 24-mer- and 20-mer-RNA-PARP-1 binding

**Protein type**	**20-mer RNA**	**24-mer RNA**
PARP1-FL	n = 1.70 ± 0.14<br>K = (2.43 ± 0.10)× 10^6 M^{-1}	n = 2.17 ± 0.15<br>K = (2.88 ± 0.23)× 10^6 M^{-1}
PARP1-ΔWGR	n = 2.13 ± 0.07<br>K = (1.46 ± 0.03)× 10^6 M^{-1}	n = 1.04 ± 0.11<br>K = (3.40 ± 0.24)× 10^6 M^{-}
PARP1-ΔCAT	n = 2.04 ± 0.13<br>K = (0.77 ± 0.05)× 10^6 M^{-1}	n = 2.77 ± 0.13<br>K = (2.91 ± 0.18)× 10^6 M^{-}
PARP1-ΔBRCT	n = 1.94 ± 0.11<br>K = (1.19 ± 0.03)× 10^6 M^{-1}	n = 2.23 ± 0.10<br>K = (2.38 ± 0.07)× 10^6 M^{-}
PARP1-ΔZn1ΔZn2	n = 2.04 ± 0.15<br>K = (1.13 ± 0.05)× 10^6 M^{-1}	n = 2.16 ± 0.19<br>K = (1.91 ± 0.11)× 10^6 M^{-1}
PARP1-ΔZn3	n = 2.17 ± 0.12<br>K = (1.30 ± 0.03)× 10^6 M^{-1}	n = 1.41 ± 0.08<br>K = (1.86 ± 0.06)× 10^6 M^{-1}

Supplemental materials and methods

Protein markers used in the SDS protein electrophoresis

We used Spectra Multicolor Broad Range Protein Ladder from ThermoFisher Scientific®. This marker gives an estimation of the size of proteins. Our observation of PARP1 running just below ~140KDa as opposed to the calculated molecular weight of ~116KDa, could be due to one or both of two things: 1. Gel conditions, which we use 4-12% Tris glycine precast PAGE gel run in MES buffer and/or 2. The Pre-Stained protein markers, gives a rough estimation and not the accurate size of the protein. We tested this last hypothesis that the Pre-Stained markers are rough estimation and indeed with other markers PARP1 does run at the predicted molecular weight of ~116KDa (see image below).

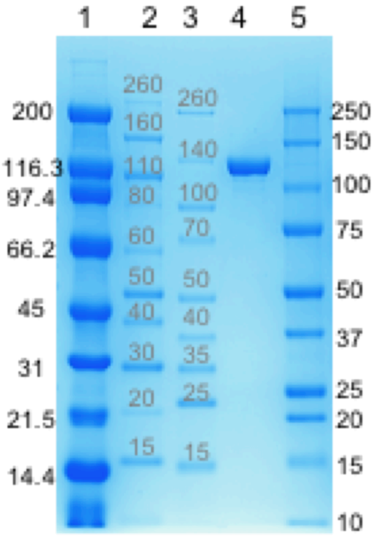

Comparative analyses of the different protein standards confirming that PARP1 runs at the correct predictive molecular weight (116KDa). However, in all our assays we use marker on Lane 3. Based on our gel conditions, western blot analyses of PARP1 in cells and recombinant, we estimated PARP1 as running about 140KDa. We now confirm that this band is PARP1, because the unstained marker in Lane 1 and Novex Sharp Pre-Stained marker, shows that PARP1 runs at 116KDa – its calculated molecular weight.

Lane 1: Bio-Rad-unstained marker (Cat# 161-0317)
Lane 2: Novex™ Sharp Pre-Stained Protein Standard (Cat #5800)
Lane 3: Spectra multicolor broad range Protein Ladder from Thermofisher Scientific (Cat #26634)
Lane 4: recombinant PARP1
Lane 5: Bio-Rad pre-stained all blue marker (Cat # 161-0373)

RNA immunoprecipitation (RIP) protocol

RIP protocol was performed according to Singh et al. 2014, with slight modifications - additional steps for nuclei extraction to eliminate the very abundant ribosomal RNAs.

Cells were resuspended in 1 X PBS and cross-linked with 0.1% of formaldehyde for 10 min at room temperature, then quenched with 3M Glycine and centrifuged to pellet the cells. Nuclei were purified by first resuspending cell pellet in cold VB buffer (30 mM Tris-HCl pH 7.5, 125 mM KCl, 5 mM $Mg(OAc)_2$, 0.15 mM spermine, 0.05 mM spermidine) and centrifuged at 250 x g for 4 min (all procedures should be done on ice at 4°C). Cell pellet was resuspended in 2.5 ml SB buffer (swelling buffer: 30 mM Tris-HCl pH 7.5, 10 mM KCl, 5 mM $Mg(OAc)_2$, 0.15 mM spermine, 0.05 mM spermidine), incubated on ice for 10 min and homogenized with 20 strokes in a 7-ml Dounce homogenizer (pestle B). The nuclear pellet was then overlaid a 2.5-ml cushion of GSB buffer (30 mM Tris-HCl pH 7.5, 10 mM KCl, 5 mM $Mg(OAc)_2$, 25% glycerol, 0.15 mM spermine, 0.05 mM spermidine) in 15-ml conical polypropylene tube and spun for 4 min at 750 x g. The nuclei pellet was then resuspended carefully in 2 ml of GSB, then 100 µl of 10% Triton-X100 was added. Samples were spun for 4 min at 750 x g and 4°C. Cell nuclie was lysed using 3 vols. of denaturing lysis buffer (20 mM Tris–HCl pH 7.5, 15 mM NaCl, 10 mM EDTA, 0.5% NP-40, 0.1% Triton X-100, 1 mM PMSF, 1/1000 volume of protease inhibitor cocktail (Epigentek), 1/250 volume of RNase inhibitor (20-40 units/µl–Sigma), 1 mM DTT, 0.1% SDS and 0.1% sodium deoxycholate). After incubation on ice for 10 min transfer in 1.5 ml tubes, samples were sonicated at 40% amplitude using 5 sec bursts with 30 sec interval a total 90 sec (18 cycles). Lysate was cleared by centrifugation, digested with DNase 1 and diluted 3-times with DLB before RIP. Nuclear lysates were pre-cleared with salmon sperm DNA/protein dynabeads for 1 hr at 4°C and incubated with PARP1/IgG antibodies overnight. RNA/antibody complexes were then precipitated with Protein A Dynabeads. Beads were washed 3-times with ice-cold denaturing wash buffer (20 mM Tris–HCl pH 7.5, 150 mM NaCl, 0.1% NP-40, 0.1% SDS and 0.1% sodium deoxycholate), 3-times with ice-cold Isotonic wash buffer (20 mM Tris–HCl pH 7.5, 150 mM NaCl, 0.1% NP-40), twice with high-stringency buffer (similar t low buffer but with 500mM NaCl). RNA was eluted in 40 ul clear sample buffer (CSB): 100 mM Tris–HCl pH 6.8, 4% SDS, 10 mM EDTA, 100 mM DTT at 25°C for 5 min and reverse crosslinked at 75°C

for 40 min, DNase1 and Proteinase K digested. RNA samples were extracted using Tizol (Invitrogen). RNA was finally precipitated and samples were further fragmented by adding 1 µl of RNA fragmentation buffer (10x) at 70°C for 4.5 min. 1 µl of the Stop solution and incubate on ice for 1 min (Ambion by life technologies; AM8740) was added to stop the reaction. RNA then was precipitated, converted to cDNA and used for RT-qPCR as described above.

Circular dichoism

Circular Dichroism. Circular dichroism (CD) was carried out at 4°C using a Jasco J-815 spectropolarimeter (JASCO analytical instruments) and a 1 mm path length CD cell (Starna Scientific). Protein concentrations at 2.5 µM in buffer CD (10 mM Tris-HCl, pH 8.0, 75 mM NaCl, 0.1 mM TCEP) were used. The final spectra for proteins represent the averages of three scans. All data were background corrected and have been converted from raw ellipticity to molar residue ellipticity ([θ]MRW) according to Equation

$$\theta \text{ (deg.cm2.dmol-1)} = \frac{\text{Ellipticity (mdeg) x}10^{6}}{\text{Pathlength(mm) x [protein](uM) x n}}$$

Where n is the number of peptide bonds in the protein and Ellipticity is the raw data from the instrument.

Primers used in RIP-RT-qPCR to validate PARP1-PAR-CLiP-seq targets

1: Chr1: 91852793-91852932

F: 5’-AAAGTTACCACAGGGATAAC-3’ R: 5’-TTCCCTATTAGTGGGTGAA-3’

2. Chr2: 230045488-230045630

F: 5’-GCCACTCCGGATTCGGGGATCT-3’ R: 5’-AACAGCAGTTGAACACGGGTC-3’3.

3. Chr1: 145277347-145277448

F: 5’-CAGAGGAAACTCTGGTG-3’ R: 5’-GGTTCAATTAGTCTTTCGCC-3’

4. Chr2: 133012685-133012798

F: 5’-GCCAGTTGGCATCGTTTATG R: 5’-AGACCAGAGCGAAAGCATTTG-3’

5. Chr21: 9827366-9827486

F: 5’-GCTGCCTTCCTTGGATGT R: 5’-CCGTGCCTACCATGGTGA-3’

6. Chr8: 70602418- 70602539

F: 5’-CTGGATAGTAGGTAGGGACAG-3’ R: 5’-CACAGGTAAATGGTGGGA-3’

Primers used to validate alternative splicing patterns after PARP1-depletion

AZI2

F: 5’-AAACAAGTTCCGTGGGACGA-3’ R: 5’-AGCTCAACTTTTCCACCTCCC-3’

BANP

F: 5’-GCCGATGTGGTTCAGATTGC-3’ R: 5’-GCGTTTCAAAGCAGGTTCGT-3’

SNHG6

F: 5’-ATGTCGCTCTTCCTTTCCCG-3’ R: 5’-AAGAACATTCATCTACAGCAACCT-3’
